# Fibulin-7 in progenitor cells promotes adipose tissue fibrosis and disrupts metabolic homeostasis in obesity

**DOI:** 10.1093/procel/pwaf084

**Published:** 2025-10-23

**Authors:** Hairong Yu, Fan Yang, Dandan Yan, Wei Chen, Lijun Yao, Hongli Chen, Siyu Lai, Jinyin Zha, Yi Sun, Yicen Zong, Jian Yu, Hong Zhang, Feng Jiang, Rong Zhang, Jian Zhang, Jing Yan, Cheng Hu

**Affiliations:** Shanghai Diabetes Institute, Shanghai Key Laboratory of Diabetes Mellitus, Shanghai Clinical Center for Diabetes, Shanghai Sixth People’s Hospital Affiliated to Shanghai Jiao Tong University School of Medicine, Shanghai 200233, China; Shanghai Diabetes Institute, Shanghai Key Laboratory of Diabetes Mellitus, Shanghai Clinical Center for Diabetes, Shanghai Sixth People’s Hospital Affiliated to Shanghai Jiao Tong University School of Medicine, Shanghai 200233, China; Shanghai Diabetes Institute, Shanghai Key Laboratory of Diabetes Mellitus, Shanghai Clinical Center for Diabetes, Shanghai Sixth People’s Hospital Affiliated to Shanghai Jiao Tong University School of Medicine, Shanghai 200233, China; Clinical Research Center, Shanghai Sixth People’s Hospital Affiliated to Shanghai Jiao Tong University School of Medicine, Shanghai 200233, China; Shanghai Diabetes Institute, Shanghai Key Laboratory of Diabetes Mellitus, Shanghai Clinical Center for Diabetes, Shanghai Sixth People’s Hospital Affiliated to Shanghai Jiao Tong University School of Medicine, Shanghai 200233, China; Shanghai Diabetes Institute, Shanghai Key Laboratory of Diabetes Mellitus, Shanghai Clinical Center for Diabetes, Shanghai Sixth People’s Hospital Affiliated to Shanghai Jiao Tong University School of Medicine, Shanghai 200233, China; Shanghai Diabetes Institute, Shanghai Key Laboratory of Diabetes Mellitus, Shanghai Clinical Center for Diabetes, Shanghai Sixth People’s Hospital Affiliated to Shanghai Jiao Tong University School of Medicine, Shanghai 200233, China; Medicinal Chemistry and Bioinformatics Center, Shanghai Jiao Tong University School of Medicine, Shanghai 200025, China; Department of Endocrinology, Affiliated Hospital of Nantong University, Nantong 226001, China; Shanghai Diabetes Institute, Shanghai Key Laboratory of Diabetes Mellitus, Shanghai Clinical Center for Diabetes, Shanghai Sixth People’s Hospital Affiliated to Shanghai Jiao Tong University School of Medicine, Shanghai 200233, China; Shanghai Key Laboratory of Regulatory Biology, Institute of Biomedical Sciences and School of Life Sciences, East China Normal University, Shanghai 200241, China; Shanghai Diabetes Institute, Shanghai Key Laboratory of Diabetes Mellitus, Shanghai Clinical Center for Diabetes, Shanghai Sixth People’s Hospital Affiliated to Shanghai Jiao Tong University School of Medicine, Shanghai 200233, China; Shanghai Diabetes Institute, Shanghai Key Laboratory of Diabetes Mellitus, Shanghai Clinical Center for Diabetes, Shanghai Sixth People’s Hospital Affiliated to Shanghai Jiao Tong University School of Medicine, Shanghai 200233, China; Shanghai Diabetes Institute, Shanghai Key Laboratory of Diabetes Mellitus, Shanghai Clinical Center for Diabetes, Shanghai Sixth People’s Hospital Affiliated to Shanghai Jiao Tong University School of Medicine, Shanghai 200233, China; Medicinal Chemistry and Bioinformatics Center, Shanghai Jiao Tong University School of Medicine, Shanghai 200025, China; Shanghai Diabetes Institute, Shanghai Key Laboratory of Diabetes Mellitus, Shanghai Clinical Center for Diabetes, Shanghai Sixth People’s Hospital Affiliated to Shanghai Jiao Tong University School of Medicine, Shanghai 200233, China; Shanghai Diabetes Institute, Shanghai Key Laboratory of Diabetes Mellitus, Shanghai Clinical Center for Diabetes, Shanghai Sixth People’s Hospital Affiliated to Shanghai Jiao Tong University School of Medicine, Shanghai 200233, China; Institute for Metabolic Disease, Fengxian Central Hospital Affiliated to Southern Medical University, Shanghai 201318, China

**Keywords:** adipose tissue fibrosis, FBLN7, TSP1, obesity, adipogenic stem and precursor cells

## Abstract

Fibrosis, resulting from excess extracellular matrix (ECM) deposition, is a feature of adipose tissue (AT) dysfunction and obesity-related insulin resistance. Emerging evidence indicates that adipogenic stem and precursor cells (ASPCs) are a crucial origin of ECM proteins and possess the potential to induce AT fibrosis. Here, we employed single-cell RNA-seq and identified a unique subset of ASPCs that were closely associated with ECM function. Within this subset, we discerned a notable upregulation in the expression of fibulin-7 (FBLN7), a secreted glycoprotein, in obese mice. Similarly, in humans, FBLN7 levels exhibited an increase in visceral fat among obese individuals and demonstrated a correlation with clinical metabolic traits. Functional studies further revealed that, in response to caloric excess, ASPC-specific *FBLN7* knockout mice displayed a diminished state of AT fibrosis inflammation, along with improved systemic metabolic health. Notably, the depletion of FBLN7 in ASPCs suppressed TGF-β-induced fibrogenic responses, whereas its overexpression amplified such responses. Mechanistically, FBLN7 interacted with thrombospondin-1 (TSP1) via its EGF-like calcium-binding domain, thereby enhancing the stability of the TSP1 protein. This, in turn, facilitated the conversion of latent TGF-β to its bioactive form, subsequently promoting TGFBR1/Smad signaling pathways. Furthermore, we developed an anti-FBLN7 neutralizing antibody, which could dramatically alleviate diet-induced AT fibrosis. These results suggest that FBLN7, produced by ASPCs, exerts a major influence in the development of AT fibrosis and may represent a potential target for therapeutic intervention.

## Introduction

As obesity progresses, adipose tissues (ATs) undergo maladaptive remodeling, characterized by adipocyte hypertrophy (Muir et al., 2016), low-grade inflammation (Mack, 2018), and continuous activation of pro-fibrotic cells, leading to fibrosis (Sun et al., 2013). As a result of excessive pathological accumulation of extracellular matrix (ECM), fibrosis of the AT impairs adipocyte hyperplasia adaptation and is recognized as a major contributor to AT dysfunction (Datta et al., 2018), obesity-associated insulin resistance (Lawler et al., 2016), as well as metabolic abnormalities, including type 2 diabetes (Lackey et al., 2014; Spencer et al., 2011). In particular, the extent of AT fibrosis may be a more important risk factor for metabolic complications than the degree of obesity itself (DeBari and Abbott, 2020). Consequently, there is an urgent need to elucidate the mechanisms underlying the development of AT fibrosis. Understanding the mechanisms mediating AT fibrosis may provide insights for developing strategies for combating obesity-associated metabolic disturbances.

The AT microenvironment, comprising multiple pro-fibrotic cells, including AT progenitors, fibroblasts, immune cells, and endothelial cells, plays a pivotal role in the progression of AT fibrosis. A recent study showed that the peptidase D (PEPD) protein, released from AT inflammatory macrophages, aggravated high-fat diet (HFD)-induced AT fibrosis and metabolic dysfunction (Pellegrinelli et al., 2022). The Hippo signaling pathway has also been found to be implicated in AT fibrosis, and the inhibition of YAP/TAZ activity in adipocytes can relieve fibrosis and improve metabolic homeostasis (Shen et al., 2022). In addition, emerging evidence suggests that platelet-derived growth factor receptor alpha (PDGFRα)-positive adipogenic stem and precursor cells (ASPCs) serve as a significant source of ECM proteins and have the capacity to cause AT fibrosis (Marcelin et al., 2017; Vila et al., 2014). During HFD-induced obesity, PDGFRα^+^CD9^high^ progenitors transition into a profibrogenic state and actively contribute to the progression of AT fibrosis (Marcelin et al., 2017). Despite considerable efforts, the precise mechanisms of PDGFRα^+^ cell-mediated AT fibrosis remain incompletely elucidated. Therefore, the identification of new regulators, specifically expressed in ASPCs and capable of regulating AT fibrosis, holds great significance.

Fibulin-7 (FBLN7), also known as TM14, is a newly identified member of the fibulin family (de Vega et al., 2007). This family comprises eight secreted ECM glycoproteins that are involved in tissue remodeling, ECM formation, and cell–matrix interactions (de Vega et al., 2009). FBLN7 has been reported to be widely expressed in the eye, teeth, placenta, and blood vessels. Moreover, it plays a vital role in biological processes, such as angiogenesis, cell morphology, cell migration, and cell adhesion. As FBLN7 can interact with ECM components, including fibronectin, integrins, and other members of the fibulin family (Chakraborty et al., 2020), it is plausible that FBLN7 could also exert a regulatory influence on tissue remodeling and fibrosis. Notably, studies have shown that *FBLN7* knockout (*FBLN7*-KO) mice were protected from renal tubular calcification and showed a trend of alleviating kidney fibrosis when fed a high-phosphate diet (Tsunezumi et al., 2018). Recently, FBLN7 has been recognized as a critical pro-fibrotic regulator of adverse cardiac remodeling after myocardial infarction by modulating the transdifferentiation process of fibroblasts into myofibroblasts (Zheng et al., 2023). Hence, it is highly probable that FBLN7 has pathogenic relevance in AT fibrosis. However, whether and how FBLN7 contributes to the progression of AT remodeling and fibrosis in obesity, along with its potential impact on metabolic disruptions, still remains unclear.

Here, we investigated the role of FBLN7 in PDGFRα^+^ ASPCs in AT fibrosis and metabolic dysfunction. By deleting or overexpressing *FBLN7* exclusively in PDGFRα^+^ cells of white adipose tissue (WAT), we elucidated the mechanism by which FBLN7 regulates AT fibrosis, systemic insulin resistance, and metabolic dysfunctions. Our findings revealed that FBLN7 activates transforming growth factor-β (TGF-β)-induced fibrogenic responses by binding to TSP1 through its epidermal growth factor (EGF)-like calcium-binding domain. Furthermore, we developed a FBLN7-neutralizing antibody, which could alleviate AT fibrosis in mice. Overall, our findings reveal that FBLN7 produced by ASPCs might represent a therapeutic target for AT fibrosis and obesity-related metabolic diseases.

## Results

### FBLN7 is upregulated in PDGFRα^+^ ASPCs of murine AT in obesity

**Figure 1.**
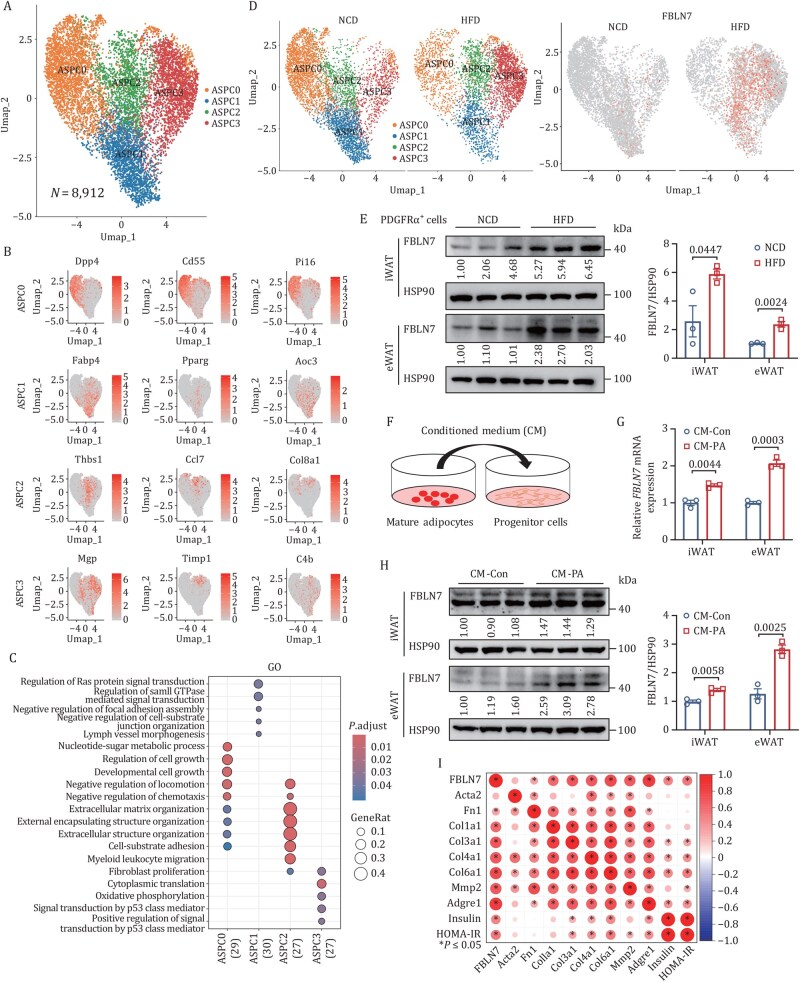
**ScRNA-seq reveals *FBLN7* upregulation in PDGFRα^+^ ASPCs of murine AT in obesity**. (A) UMAP plot of scRNA-seq for four clusters of ASPCs from the merged eWAT SVF of NCD and HFD mice. (B) UMAP plots of the normalized expression of representative marker genes for ASPC clusters. (C) GO enrichment analysis of ASPC clusters using the top 30 marker genes. (D) UMAP plots of scRNA-seq for four clusters of ASPCs from NCD and HFD mice (left), and *FBLN7* expression in UMAP (right). (E) Western blot and quantification of FBLN7 in PDGFRα^+^ SVF cells from WAT of NCD or HFD mice (*n *= 3). (F–H) CM of differentiated white mature adipocytes was collected to treat progenitor cells. (F) Illustration of the experiment. (G) RT-qPCR of *FBLN7* mRNA expression in progenitor cells (*n *= 3). (H) Western blot and quantification of FBLN7 in progenitor cells (*n *= 3). (I) Pearson correlations of *FBLN7* expression with pro-fibrotic markers, pro-inflammatory markers of iWAT, and metabolic parameters in HFD-fed mice (0–19 weeks, *n *= 60). Data are shown as mean ± SEM (E, G, and H). *P*-values were determined by unpaired two-tailed Student’s *t*-test (E, G, and H).

To identify new regulators in the development of obesity-related AT fibrosis, we first generated a comprehensive atlas of cell plasticity in WAT at single-cell resolution in response to obesity. This was achieved by performing single-cell RNA sequencing (scRNA-seq) on the stromal vascular fractions (SVFs) of epididymal WAT (eWAT) from normal chow diet (NCD)- and HFD-fed male *C57BL*/*6J* mice. Unsupervised clustering analysis of 14,145 quality control-positive cells from both NCD- and HFD-fed mice divided the cells into 12 distinct clusters (Fig. S1A). Further analysis of ASPCs marked by PDGFRα revealed four ASPC clusters (ASPC 0–3), based on their gene signatures and specific marker genes (Fig. 1A and 1B). By mapping our datasets with the recent eWAT scRNA-seqs (Merrick et al., 2019; Nahmgoong et al., 2022), *Dpp4*, *Cd55*, and *Pi16*-expressing ASPC0 was annotated as “multipotent progenitors”; the high-adipogenic capacity fraction, ASPC1, was referred to “committed preadipocytes”; ASPC3, abundantly expressing *Mgp* and *Timp1*, appeared to be “adipogenesis-regulatory cells (Aregs)” that have been reported to suppress adipogenesis (Schwalie et al., 2018). Nevertheless, despite sharing a gene signature with ASPC3 in part, ASPC2, dominantly and exclusively expressing ECM-related genes, was previously unrecognized and thus gained our attention. Gene Ontology (GO) and Kyoto Encyclopedia of Genes and Genomes (KEGG) analyses also indicated that pathways related to ECM function were markedly enriched in ASPC2 (Figs. 1C and S1B). In support of this, pseudotime trajectories analysis predicted that “multipotent progenitors” ASPC0 had two distinct developmental trajectories: one leading to ASPC1 preadipocytes (cell fate 1) and the other to ASPC3 Aregs (cell fate 2). In contrast, ASPC2 did not exhibit any specific direction (Fig. S1C). Taken together, ASPC2 appeared to represent a unique cell type contributing to ECM function, suggesting it might be involved in AT fibrosis.

Given that HFD evoked ECM-related pathways in ASPC2 compared with the NCD condition (Fig. S1D), we proceeded to analyze the differentially expressed genes (DEGs) in ASPC2 between the NCD- and HFD-fed mice to identify the “standout” genes that contribute to obesity-induced AT fibrosis. The top DEGs showing the most significant effects (according to the adjusted *P *< 1 × 10^−50^) were first selected and then ranked by the value of pct. HFD/pct.NCD (defined as the ratio of the percentage of cells expressing gene levels in HFD to those in NCD). Among the top genes, *FBLN7*, which was expressed in 8-fold more cells under HFD than in the condition of NCD, emerged as a notable candidate (Figs. 1D and S1E). Strikingly, *FBLN7* was almost exclusively expressed in ASPC2 (Fig. S1F). In summary, *FBLN7* expression level underwent a substantial upregulation in the ASPC2 cluster of murine SVF PDGFRα^+^ cells upon HFD challenge.

Subsequently, we verified the findings from the scRNA-seq dataset. Compared with their lean counterparts, mRNA levels of *FBLN7* in both inguinal WAT (iWAT) and eWAT were significantly increased in mice with diet-induced obesity (DIO) or genetic obesity caused by leptin (*ob*/*ob*) or leptin receptor (*db*/*db*) deficiency (Fig. S2A). Immunohistochemical (IHC) staining and Western blotting also demonstrated the same upward trend in FBLN7 expression (Fig. S2B and S2C). By further separating the SVF and the mature adipocyte fraction (MAF) from WAT, we found that obesity enhanced FBLN7 expression significantly in the SVF (Fig. S2D). Leptin (Lep) and CD45 were used as controls for MAF and SVF, respectively (Yan et al., 2022). In addition, considering that over 95% of adherent SVF cells were PDGFRα^+^ (Fig. S2E), we cultured these cells from both NCD- and HFD-fed mice and confirmed that HFD resulted in a dramatic upregulation of *FBLN7* expression in PDGFRα^+^ ASPCs (Fig. 1E). To further explore the HFD-associated factors that might trigger FBLN7 expression, we established an *in vitro* model using a panel of stimulators, including palmitic acid (PA), tumor necrosis factor alpha (TNFα), interleukin 1 beta (IL-1β), and lipopolysaccharide (LPS). These stimulators were individually applied to stimulate the adherent PDGFRα^+^ progenitor cells and mature adipocytes. The results showed that only the conditioned medium (CM) from PA-treated mature adipocytes led to a significant increase in the abundance of FBLN7 in progenitor cells (Figs. 1F-H, S2F and S2G). These findings indicate that cytokines produced by adipocytes contributed to the FBLN7 upregulation in response to HFD challenge.

Considering the role of the ASPC2 cluster involved in the ECM pathway, ATs from mice fed with HFD for 0–19 weeks were collected to test the link between FBLN7 and fibro-inflammation. The results revealed that the expression levels of FBLN7 commenced a significant increase starting from the sixth week of the HFD (Fig. S2H), suggesting that FBLN7 induction was an early driver in obesity development, rather than a late downstream amplifier. Correlation matrix analysis showed that *FBLN7* mRNA levels in iWAT were positively correlated with the mRNA levels of fibro-inflammation markers such as *Fn1*, *Col1a1*, *Col3a1*, *Col4a1*, *Col6a1*, *Mmp2*, and *Adgre1*. Moreover, *FBLN7* levels also exhibited a positive correlation with serum fasting insulin levels, and systemic insulin resistance measured by the ‌homeostatic model assessment of insulin resistance (HOMA-IR) (Fig. 1I). Altogether, these results suggest that FBLN7 in ASPCs may couple fibro-inflammation and metabolic dysfunction.

### Human FBLN7 expression correlates with metabolic traits

**Figure 2.**
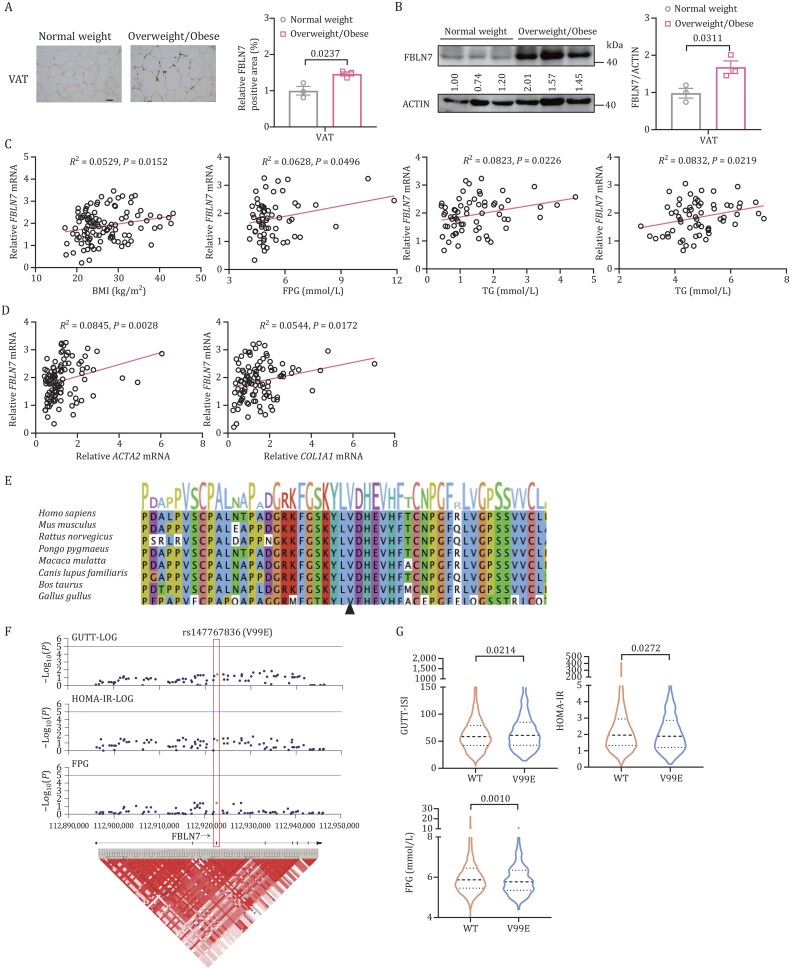
**Human FBLN7 expression correlates with metabolic traits**. (A) Representative IHC staining and quantification of FBLN7 in visceral fat from overweight/obese and normal-weight individuals (*n *= 3). Scale bars, 50 µm. (B) Western blot and quantification of FBLN7 in visceral fat from overweight/obese and normal-weight individuals (*n *= 3). (C) Correlation between *FBLN7* mRNA expression in human visceral fat and clinical metabolic traits: BMI (*n *= 111), FPG (*n *= 62), TG (*n *= 63), and TC (*n *= 63). *FBLN7* mRNA levels were normalized to *RPLP0* mRNA. (D) Correlation between *FBLN7* mRNA expression and fibrotic marker genes *ACTA2* and *COL1A1* in human visceral fat (*n *= 104). (E) Conservation analysis of FBLN7 amino acid residue at position 99 across species. (F) Region plot of genetic associations between *FBLN7* gene variants with GUTT-ISI, HOMA-IR, and FPG. Genome build is aligned with the GRCh37/hg19 assembly. (G) Levels of GUTT-ISI, HOMA-IR, and FPG in *FBLN7* WT and V99E variant carriers. Data were log-transformed before statistical analysis. Data are presented as mean ± SEM (A and B). Violin plots (G) show the median value, along with 25th and 75th percentiles. *P* values were determined by unpaired two-tailed Student’s *t*-test (A, B, and G). Pearson’s correlation analysis, showing *R*^2^ and *P* values, was performed for (C) and (D). (F) Linear regression analysis under the additive genetic model was used to test the effects of *FBLN7* variants on metabolic traits, adjusting for sex and age.

To further investigate whether FBLN7 experiences upregulation in human obesity, we analyzed its expression in visceral fat biopsies from a cohort of individuals with a wide body mass index (BMI) range (Table S1). IHC staining and Western blotting revealed that overweight or obese individuals (BMI ≥ 24) had remarkably higher expression of FBLN7 in visceral fat when compared with those with normal BMI (BMI < 24) (Fig. 2A and 2B). Moreover, we observed a positive correlation between *FBLN7* mRNA expression of visceral fat and clinical quantitative traits indicating obesity and glucose metabolism, including BMI, fasting plasma glucose (FPG), as well as lipid metabolism indicators such as triglyceride (TG) and total cholesterol (TC) (Fig. 2C). In addition, *FBLN7* expression also demonstrated a positive correlation with pro-fibrosis markers such as *ACTA2* and *COL1A1* in visceral fat (Fig. 2D), emphasizing its potential role in AT fibrosis.

We also delved into the association of *FBLN7* genetic variants (single-nucleotide polymorphisms [SNPs]) with metabolic traits in humans using microarray genotyping datasets from the Shanghai Nicheng Cohort Study (Chen et al., 2018). Notably, we identified a missense variant p.Val99Glu (c.296T>A, rs147767836) (V99E). Conservation analysis showed that amino acid site 99 of FBLN7 is highly conserved across common species, such as *Homo sapiens* and *Mus musculus* (Fig. 2E). The genotype distribution was 10,708 subjects (97.21%) with TT, 306 subjects (2.78%) with TA, and only 1 subject (0.0091%) with AA. The baseline characteristics of participants with different genotypes were summarized in Table S2. Multiple linear regression adjusting for age and sex showed that rs147767836-A allele was associated with the Gutt insulin sensitivity index (GUTT-ISI), HOMA-IR, and FPG (Fig. 2F). Individuals carrying the V99E variant exhibited a mild yet statistically significant improvement in insulin sensitivity as evidenced by GUTT-ISI and HOMA-IR, and a reduction in FPG levels (Fig. 2G).

Collectively, these human data indicate that FBLN7 is closely correlated with metabolic traits, emphasizing its critical role in metabolic dysfunction.

### Global FBLN7 ablation alleviates insulin resistance and metabolic dysfunction

To clarify the role of FBLN7 in metabolic homeostasis, we examined the phenotypic characteristics of mice with global knockout of *FBLN7* (KO, *FBLN7*^−/−^) in comparison to their age- and sex-matched wild-type (WT, *FBLN7*^+/+^) littermates. The ablation of FBLN7 was confirmed in the KO mice (Fig. S3A). Consistent with previous reports (Tsunezumi et al., 2018), these KO mice did not show any obvious developmental or health defects (Fig. S3B). Following a 20-week HFD challenge, the body weights of the KO mice were found to be comparable to those of WT control mice (Fig. S3C). Additionally, no significant histological differences in adipocyte size were observed between the WT and KO genotypes (Fig. S3D and S3E). However, the eWAT of KO mice exhibited a higher weight (Fig. S3F) and a greater number of adipocytes in comparison to the controls (Fig. S3G), indicating a hyperplastic expansion of eWAT. Moreover, compared with control mice, the HFD KO mice displayed elevated serum adiponectin levels (Fig. S3H).

Next, we assessed glucose homeostasis and lipid metabolism. Compared with WT mice, KO mice had lower fasting insulin levels (Fig. S3I) and showed improved glucose tolerance and insulin sensitivity (Fig. S3J and S3K). In addition, in concordance with lower liver weights and intrahepatic TG contents (Fig. S3L and S3M), hematoxylin and eosin (H&E) and Oil Red O staining indicated reduced lipid accumulation in KO mice (Fig. S3N), suggesting that *FBLN7* ablation protected mice from HFD-induced hepatic steatosis. Therefore, it can be inferred that *FBLN7* ablation may mitigate HFD-induced metabolic dysfunction in mice, including insulin resistance, dysregulation of glycolipid metabolism, and liver steatosis.

### FBLN7 expressed in ASPCs regulates systemic metabolic homeostasis

Given that FBLN7 is predominantly localized in ASPCs in obese ATs, we further generated ASPC-specific KO mice using the PDGFRα Cre-lox system to investigate the role of FBLN7 in ASPCs during the pathogenesis of metabolic disorders. We intercrossed mice carrying a conditional loxP flanked (“floxed”) allele of *FBLN7* (also named as *FBLN7*-Flox mice, used as controls) with the *PDGFRα*-Cre mice to create ASPC-specific *FBLN7*-KO (referred to hereafter as *FBLN7*-APKO) mice. Our analysis confirmed that FBLN7 was successfully ablated in ASPCs from WAT (Fig. S4A and S4B). FBLN7 expression was also reduced in the eWAT and iWAT of *FBLN7*-APKO mice compared to those of *FBLN7*-Flox mice, while there were slight or insignificant changes in other tissues, thereby validating the specificity of the APKO mice (Fig. S4C and S4D). Notably, the APKO mice were born healthy and fertile, and did not show any gross abnormalities (Fig. S4E).

**Figure 3.**
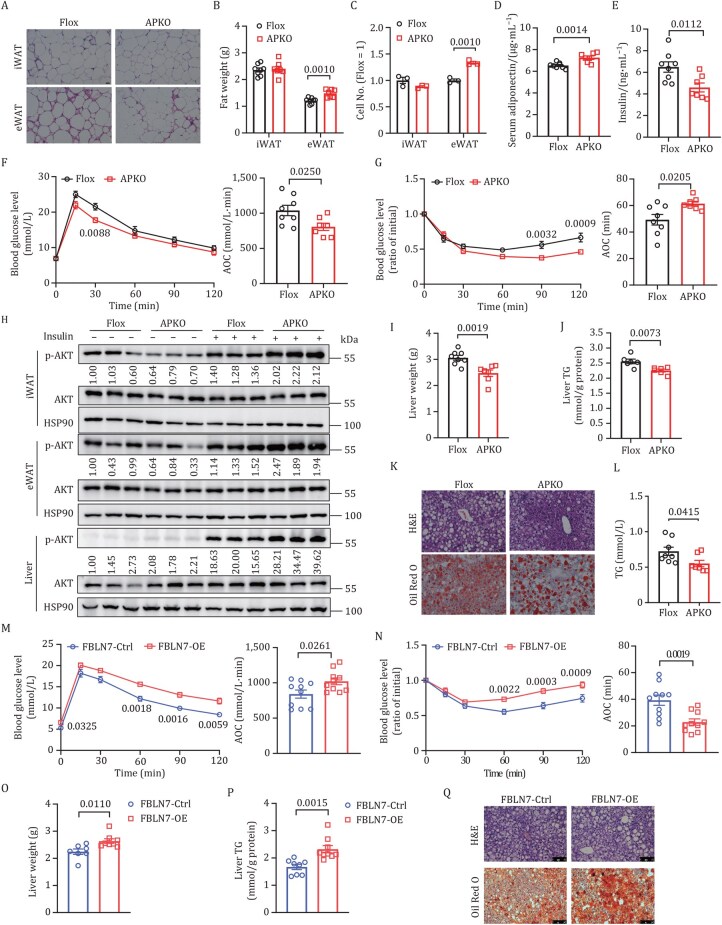
**FBLN7 expressed in ASPCs regulates systemic metabolic homeostasis**. Male *FBLN7*-Flox and age-matched *FBLN7*-APKO mice were fed HFD at 7 weeks of age and maintained for 25 weeks (A–L). (A) Representative H&E images of WAT. Scale bars, 25 µm. (B) Fat-pad weights of Flox (*n *= 8) and APKO (*n *= 7) mice. (C) Quantification of adipocyte cell numbers (*n *= 3). (D) Serum adiponectin levels (*n *= 8, Flox mice; *n *= 7, APKO mice). (E) Serum insulin levels (*n *= 8, Flox mice; *n *= 7, APKO mice). (F) GTT and AOC (*n *= 8, Flox mice; *n *= 7, APKO mice). (G) ITT and AOC (*n *= 8, Flox mice; *n *= 7, APKO mice). (H) Western blot of AKT phosphorylation in iWAT, eWAT, and liver after insulin or saline administration (*n *= 3). (I) Liver weight (*n *= 8, Flox mice; *n *= 7, APKO mice). (J) Quantification of hepatic triglycerides (*n *= 6). (K) Representative images of H&E (top) and Oil Red O (bottom) staining of the liver. Scale bars, 25 µm. (L) Serum TG levels (*n *= 8, Flox mice; *n *= 7, APKO mice). Male iWAT (8 weeks old) received bilateral iWAT injections of AAV-*FBLN7* or AAV-*Control* and were fed HFD for 25 weeks (M–Q). (M) GTT and AOC (*n *= 10). (N) ITT and AOC (*n *= 10). (O) Liver weight (*n *= 7, *FBLN7*-Ctrl mice; *n *= 8, *FBLN7*-OE mice). (P) Quantification of hepatic triglycerides (*n *= 8, *FBLN7*-Ctrl mice; *n *= 9, *FBLN7*-OE mice). (Q) Representative images of H&E (top) and Oil Red O (bottom) staining of the liver. Scale bars, 100 µm. Data are shown as means ± SEM (B–G, I, J, and L–P). For statistical analysis, unpaired two-tailed Student’s *t*-test was performed for (B–G, I, J, and L–P), and a two-way ANOVA with Sidak’s multiple comparison test was performed for (F, G, M, and N).

Similar to the global KO mice, *FBLN7*-APKO mice showed minimal changes in body weight (Fig. S4F) and adipocyte cell size (Figs. 3A and S4G). The administration of the HFD resulted in increased eWAT hyperplasia in the *FBLN7*-APKO mice when compared to control littermates (Fig. 3B and 3C), which is in line with the notion that shifting AT expansion from hypertrophy to hyperplasia may prevent pathological remodeling and AT dysfunction. *FBLN7*-APKO mice also displayed higher serum levels of adiponectin (Fig. 3D). In addition, *FBLN7* deletion in ASPCs improved HFD-induced hyperinsulinemia, glucose dysregulation, and insulin resistance (Fig. 3E-G). Insulin-stimulated AKT phosphorylation was also significantly higher in the iWAT, eWAT, and liver of *FBLN7*-APKO mice than that of the *FBLN7*-Flox mice (Figs. 3H and S4H). However, no significant differences were observed in muscle (Fig. S4I), suggesting that the partial knockdown (KD) of FBLN7 in muscle did not contribute to systemic metabolic performance. Regarding lipid metabolism, *FBLN7*-APKO mice exhibited reduced lipid accumulation in the liver (Fig. 3I-K), along with lower serum levels of TG, TC, and non-esterified fatty acid (NEFA) (Figs. 3L, S4J and S4K) when compared to *FBLN7*-Flox mice. These data indicate that FBLN7 deficiency in ASPCs leads to phenotypic effects that closely resemble those observed in global KO mice.

To further validate the effects of the FBLN7 on metabolic deterioration, we constructed a local *FBLN7* overexpression model (*FBLN7*-OE) by injecting adeno-associated virus (AAV)-*FBLN7* into the iWAT of 8-week-old WT *C57BL/6J* mice. The successful overexpression of FBLN7 in ASPCs was validated (Fig. S5A and S5B). *FBLN7*-OE and control mice were subjected to an HFD for 25 weeks. In contrast to *FBLN7*-APKO mice, *FBLN7*-OE mice showed lower adiponectin levels and higher fasting insulin levels (Fig. S5C and S5D). In addition, *FBLN7*-OE mice exhibited impaired glucose tolerance (Fig. 3M), as well as reduced insulin sensitivity, as revealed by the insulin tolerance test (Fig. 3N) and reduced AKT phosphorylation in iWAT and liver relative to controls (Fig. S5E). The overexpression of FBLN7 in iWAT also exacerbated liver steatosis (Fig. 3O–Q) and increased serum TG, TC, and NEFA levels (Fig. S5F and S5H). Collectively, these data indicate that FBLN7 may play a pivotal role in exacerbating metabolic disorders.

### FBLN7 expressed in ASPCs modulates obesity-related AT fibrosis

**Figure 4.**
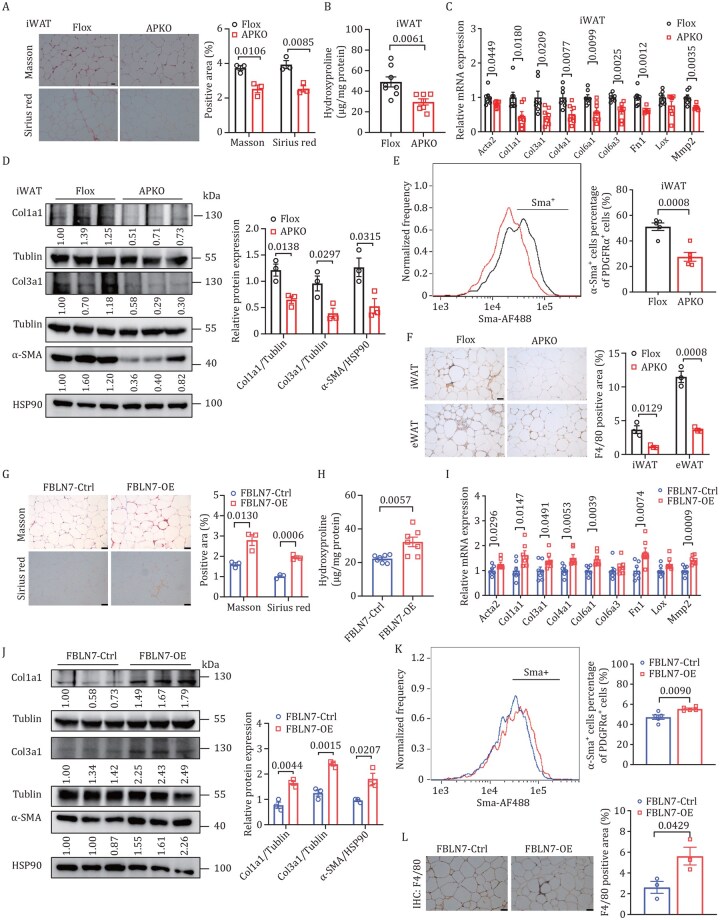
**FBLN7 expressed in ASPCs modulates obesity-related AT fibrosis *in vivo***. Male *FBLN7*-Flox and age-matched *FBLN7*-APKO mice were fed HFD for 25 weeks (A–F). (A) Representative Masson’s trichrome and Sirius Red staining images and quantitative analysis in iWAT (*n *= 3). Scale bars, 25 µm. (B) Hydroxyproline content in iWAT (*n *= 8, Flox mice; *n *= 7, APKO mice). (C) RT-qPCR indicating the mRNA abundance of pro-fibrosis genes in iWAT (*n *= 7). (D) Western blot and quantification of pro-fibrosis protein in iWAT (*n *= 3). (E) Representative flow cytometry analysis and quantification of Sma^+^ cells within PDGFRα^+^ cells from iWAT (*n *= 5). (F) Representative F4/80 IHC staining and quantitative analysis in WAT (*n *= 3). Scale bars, 50 µm. *FBLN7-*OE and control mice were fed HFD for 25 weeks (G–L). (G) Representative Masson’s trichrome and Sirius Red staining images and quantitative analysis in iWAT (*n *= 3). Scale bars, 50 µm. (H) Hydroxyproline contents of iWAT (*n *= 7). (I) RT-qPCR indicating the mRNA abundance of pro-fibrosis genes in iWAT (*n *= 7). (J) Western blot and quantification of pro-fibrosis protein in iWAT (*n *= 3). (K) Representative flow cytometry analysis and quantification of Sma^+^ cells within PDGFRα^+^ cells from iWAT (*n *= 5). (L) Representative F4/80 IHC staining and quantitative analysis of iWAT (*n *= 3). Scale bars, 50 µm. Data are shown as mean ± SEM. Two-tailed Student’s *t*-test was performed.

Given that AT fibrosis is a pathological process of AT remodeling that can control systemic metabolic homeostasis, and FBLN7 is considered involved in the ECM function, we embarked on a further exploration to determine whether the effects of FBLN7 in ASPCs on metabolic homeostasis were coupled to AT fibrosis. Following an HFD for 25 weeks, *FBLN7*-APKO mice exhibited a markedly decrease in ECM accumulation in WAT, as revealed by Masson’s trichrome and Sirius red staining (Figs. 4A and S6A). Parallel trends were observed in the hydroxyproline levels (Figs. 4B and S6B) as well as in the expression levels of fibrotic markers (Figs. 4C, 4D, S6C and S6D). In line with these results, flow cytometry analysis revealed a significant reduced proportion of α-Sma^+^ cells within PDGFRα^+^ populations in WAT (Figs. 4E and S6E) of *FBLN7*-APKO mice compared to their *FBLN7*-Flox littermates. The gating strategies employed and the negative control results were shown in Fig. S6F and S6G, respectively. These results confirmed a diminished AT fibrosis environment in *FBLN7*-APKO mice. It was well documented that extensive AT fibrosis is typically accompanied by AT inflammation (Crewe et al., 2017). We found that *FBLN7* depletion in ASPCs reduced HFD-induced F4/80^+^ crown-like structures (CLS) in WAT (Fig. 4F). Similar to male mice, female APKO mice exhibited metabolic phenotypes comparable to those of male mice, including improved metabolic health (Fig. S7A-I) and alleviated AT fibrosis (Fig. S7J-N).

In contrast, *FBLN7*-OE mice displayed a significant elevation in ECM accumulation (Fig. 4G), hydroxyproline levels (Fig. 4H), and expression of pro-fibrosis markers in iWAT (Fig. 4I and 4J). In parallel, *FBLN7* overexpression resulted in an increase in the fraction of α-Sma^+^ cells within the PDGFRα^+^ population from iWAT (Fig. 4K). The infiltration of F4/80^+^ macrophages was also substantially augmented in *FBLN7*-OE mice, indicating increased AT inflammation (Fig. 4L).

Furthermore, to overexpress *FBLN7* specifically in PDGFRα^+^ progenitor cells *in vivo*, we administered AAV-double-floxed inverted orientation (DIO)-*FBLN7* virus into the iWAT of *PDGFRα*-Cre mice at the age of 8 weeks, thereby generating DIO-*FBLN7*-OE mice. The specific overexpression of FBLN7 in PDGFRα^+^ progenitor cells was confirmed (Fig. S8A). Similar to *FBLN7*-OE mice, DIO-*FBLN7*-OE mice also displayed disrupted metabolic homeostasis (Fig. S8B–I) and increased AT fibrosis and inflammation (Fig. S8J–O). These data demonstrate that the phenotypes of *FBNL7*-OE mice are predominantly attributed to the PDGFRα^+^ progenitor cells.

**Figure 5.**
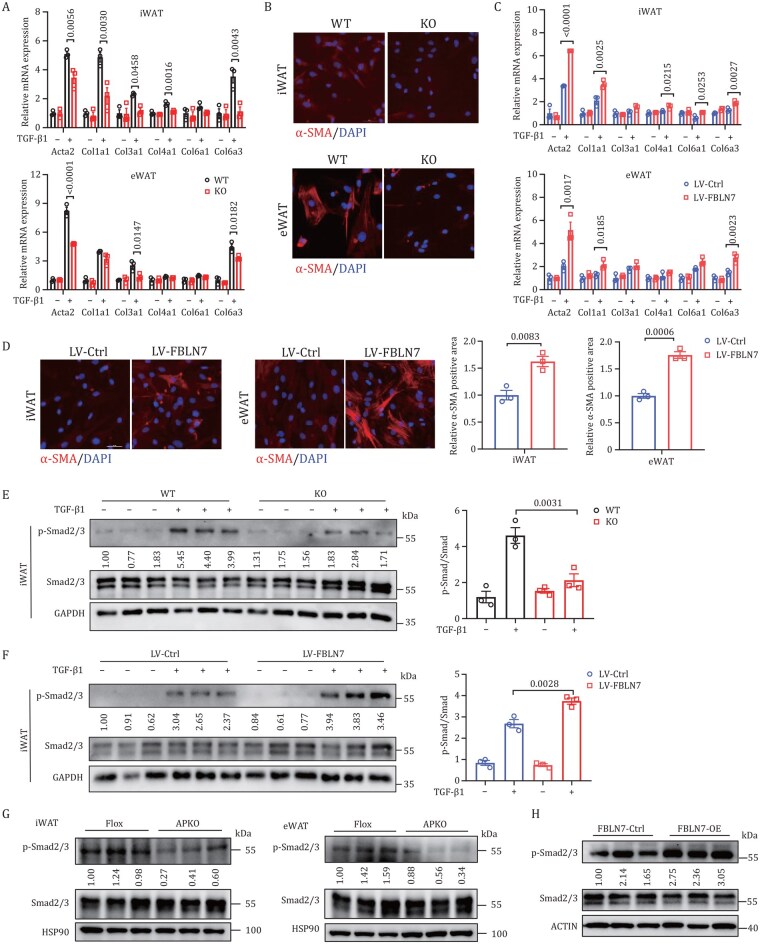
**FBLN7 modulates ASPC fibrosis processes *in vitro***. The progenitor cells were obtained from iWAT and eWAT of WT and KO mice aged 6–8 weeks, and then treated with TGF-β1 (A and B). (A) RT-qPCR indicating mRNA abundance of pro-fibrosis genes (*n *= 3). (B) Representative immunofluorescence images of α-SMA staining (red) (*n *= 3). Nuclei were stained with DAPI (blue). Scale bars, 100 µm. The progenitor cells were obtained from *C57BL*/*6J* mice, and then infected with LV-*Ctrl* or LV-*FBLN7* (C and D). (C) RT-qPCR indicating mRNA abundance of pro-fibrosis genes (*n *= 3). (D) Representative immunofluorescence images and quantitative analysis of α-SMA staining (red) (*n *= 3). Nuclei were stained with DAPI (blue). Scale bars, 100 µm. Western blot and quantification of p-Smad2/3 and Smad2/3 in iWAT cells from WT and KO mice (E) (*n *= 3), and in cells infected with LV-*Ctrl* or LV-*FBLN7* (F) (*n *= 3). Western blot of p-Smad2/3 and Smad2/3 in iWAT and eWAT from Flox and APKO mice (G) (*n *= 3), and in iWAT from *FBLN7*-Ctrl and *FBLN7*-OE mice (H) (*n *= 3). Data are shown as mean ± SEM. Two-tailed Student’s *t*-test was performed for (D). One-way ANOVA with Tukey’s multiple comparison test was performed for (A, C, E, and F).

Having established the critical role of FBLN7 in inducing AT fibrosis *in vivo*, we were driven to further substantiate its effects through *in vitro* experiments. TGF-β is the master regulator of fibrosis, which promotes ECM accumulation through the phosphorylation of Smad2 and Smad3 (Meng et al., 2016). Besides, the gene set variation analysis (GSVA) for KEGG of our scRNA-seq dataset showed the TGF-β signaling pathway was activated in ASPC2 (Fig. S9A). Therefore, we isolated SVF cells from WAT, cultured ASPCs, and then stimulated these cells using TGF-β1 to observe TGF-β-induced fibrosis. To achieve *FBLN7* ablation in ASPCs (Fig. S9B and S9C), we isolated ASPCs from WT and KO mice. Notably, we observed no significant difference in the number of PDGFRα^+^ progenitor cells between these two groups (Fig. S9D). As expected, upon TGF-β1 stimulation, the expression level of pro-fibrosis markers was markedly lower in *FBLN7*-KO cells than in WT ones (Fig. 5A). Immunofluorescence staining revealed a substantial reduction (78.78% reduction in iWAT and 76.04% reduction in eWAT) in the α-SMA protein in *FBLN7*-KO ASPCs (Figs. 5B and S9E), indicating alleviated ASPC fibrosis. In contrast, lentivirus (LV)-mediated *FBLN7* overexpression in ASPCs (Fig. S9F and S9G) promoted pro-fibrosis responses, as indicated by substantially increased mRNA levels of pro-fibrosis markers (Fig. 5C) and increased (53.82% increase in iWAT and 57.34% increase in eWAT) α-SMA protein levels (Fig. 5D). Furthermore, *FBLN7* KO strongly inhibited the phosphorylation of Smad2/3 (Figs. 5E and S9H), which mediates TGF-β/Smad signaling and the subsequent fibrosis cascade. In contrast, *FBLN7* overexpression in ASPCs increased TGF-β/Smad signaling (Figs. 5F and S9I).

In addition, we observed that the phosphorylation levels of Smad2/3 were significantly decreased in WAT of *FBLN7*-APKO mice compared to the controls (Figs. 5G and S9J). Conversely, *FBLN7* overexpression in iWAT activated TGF-β/Smad signaling through increased phosphorylation of Smad2/3 (Figs. 5H and S9K). Collectively, these data indicate that FBLN7 in ASPCs exerts a regulatory influence on fibrosis through the TGF-β/Smad signaling pathway.

Besides, the progenitor cells showed no change in their proliferation capacity when *FBLN7* was knocked down or overexpressed using lentivirus, as displayed by Cell Counting Kit-8 (CCK8) (Fig. S10A and S10B) and EdU assays (Fig. S10C and S10D). Given that the *PDGFRα*-Cre line gives rise to all mature adipocytes in all WAT depots (Berry and Rodeheffer, 2013), we also explored whether FBLN7 affected the differentiation of PDGFRα^+^ progenitor cells or the physiologic function of mature adipocytes. Our findings indicated that neither knocking down nor overexpressing *FBLN7* in PDGFRα^+^ cells and mature white adipocytes had any effect on adipogenic capacity, evidenced by comparable levels of adipogenic markers, as well as Oil Red O staining (Fig. S10E–J). Moreover, FBLN7 also had no obvious impact on the thermogenic capacity of mature beige adipocytes (Fig. S10K and S10L). These results suggest that FBLN7 has a relatively limited functional influence on adipocytes.

### FBLN7 mediates pro-fibrosis signaling via TSP1

**Figure 6.**
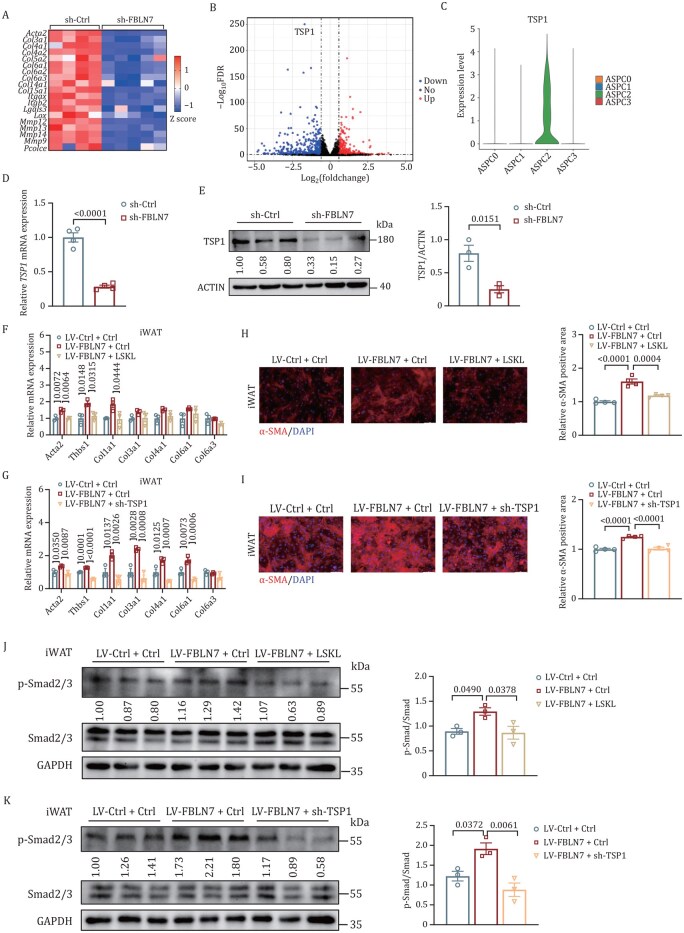
**FBLN7 mediates pro-fibrosis signaling via TSP1 *in vitro***. The progenitor cells of iWAT were infected with LV-sh-*Ctrl* (*n *= 4) or LV-sh-*FBLN7* (*n *= 5), followed by TGF-β1 stimulation and subsequent RNA-seq analysis (A and B). (A) Expression profiles of pro-fibrosis genes. (B) Volcano plot of gene expression analysis. (C) Violin plots showing *TSP1* expression in ASPC clusters by scRNA-seq analysis. (D) RT-qPCR of *TSP1* mRNA expression in iWAT cells infected with LV-sh-*Ctrl* or LV-sh-*FBLN7* (*n *= 4). (E) Western blot and quantification of TSP1 in iWAT cells infected with LV-sh-*Ctrl* or LV-sh-*FBLN7* (*n *= 3). RT-qPCR indicating mRNA abundance of pro-fibrosis genes in iWAT cells infected with LV-*Ctrl* or LV-*FBLN7*, followed by LSKL treatment (F) (*n *= 3), or TSP1 knockdown via LV-sh-*TSP1* (G) (*n *= 3). Representative immunofluorescence images and quantitative analysis of α-SMA staining (red) in LV-*Ctrl* or LV-*FBLN7* iWAT cells after LSKL treatment (H) (*n *= 4), or TSP1 knockdown (I) (*n *= 4). Nuclei were stained with DAPI (blue). Scale bars, 100 µm. Western blot and quantification of p-Smad2/3 and Smad2/3 in LV-*Ctrl* or LV-*FBLN7* iWAT cells after LSKL treatment (J) (*n *= 3), or TSP1 knockdown (K) (*n *= 3). Data are presented as mean ± SEM. Unpaired two-tailed Student’s *t*-test was performed for (D and E). One-way ANOVA with Tukey’s multiple comparison tests was performed for (F–K).

We proceeded to explore the molecular mechanisms by which FBLN7 regulates the TGF-β/Smad signaling pathway in ASPCs. To determine the downstream signals and target genes of FBLN7, we conducted RNA-seq transcriptomic analyses (Fig. S11A and S11B) on WT ASPCs infected with lentiviral short hairpin RNA (shRNA) specific for *FBLN7* (sh-*FBLN7*) versus those infected with control lentivirus (sh-*Ctrl*) after TGF-β1 stimulation. The gene set enrichment analysis (GSEA) and GO analysis showed that the ECM-related pathway was significantly downregulated in the sh-*FBLN7* group (Fig. S11C and S11D). As expected, a substantial number of genes involved in ECM development exhibited reduced expression levels in the sh-*FBLN7* group (Fig. 6A). A detailed analysis (false discovery rate [FDR] < 0.05) identified a total of 864 DEG-363 upregulated and 501 downregulated genes. Notably, the gene *TSP1*, encoding thrombospondin-1 (TSP1), emerged as the most significantly downregulated gene in the sh-*FBLN7* group (Fig. 6B), which was congruent with the result from our scRNA-seq datasets that *TSP1* stood out as the marker gene of ASPC2 (Fig. 6C). Subsequently, we confirmed that *TSP1* mRNA and protein levels were significantly reduced in *FBLN7* KD ASPCs (Fig. 6D and 6E). Additionally, we examined the TSP1 expression levels in ATs of different mouse models and found, as anticipated, a decrease in TSP1 expression in both KO (Fig. S11E and S11F) and APKO mice (Fig. S11G and S11H), whereas an elevation in *FBLN7*-OE mice (Fig. S11I and S11J).

TSP1, a matricellular and secreted protein, has been reported to accelerate fibrotic responses by activating latent TGF-β1 (Murphy-Ullrich and Suto, 2018) and triggering downstream Smad signaling (Anastasi et al., 2020). Moreover, TSP1 expression is elevated upon activation of TGF-β/Smad signaling (Daubon et al., 2019; Joseph et al., 2022), suggesting the existence of a positive feedback loop between TSP1 and the TGF-β/Smad pathway. Considering the significant role of FBLN7 in modulating TGF-β signaling, we further investigated whether TSP1 functioned as a downstream target of FBLN7 in the process of mediating TGF-β/Smad signaling and the subsequent fibrosis development. We used the Leu-Ser-Lys-Leu (LSKL) peptide, a peptide antagonist of TSP1, to inhibit TSP1-mediated activation of TGF-β in vitro. Remarkably, treatment with LSKL led to a substantial reversal of fibrosis in cells overexpressing FBLN7, as evidenced by the changes in pro-fibrosis gene expression (Figs. 6F and S12A). Furthermore, *TSP1* KD using lentiviral sh-RNA (sh-*TSP1*) effectively inhibited the inducible effect of FBLN7 on fibrosis (Figs. 6G and S12B). Immunofluorescence staining revealed that both LSKL and *TSP1* KD decreased α-SMA levels, which were elevated by *FBLN7* overexpression (Figs. 6H, 6I, S12C and S12D). In addition, the enhanced phosphorylation of Smad2/3 observed in cells overexpressing *FBLN7* was reversed by LSKL as well as sh-*TSP1* (Figs. 6J, 6K, S12E and S12F).

**Figure 7.**
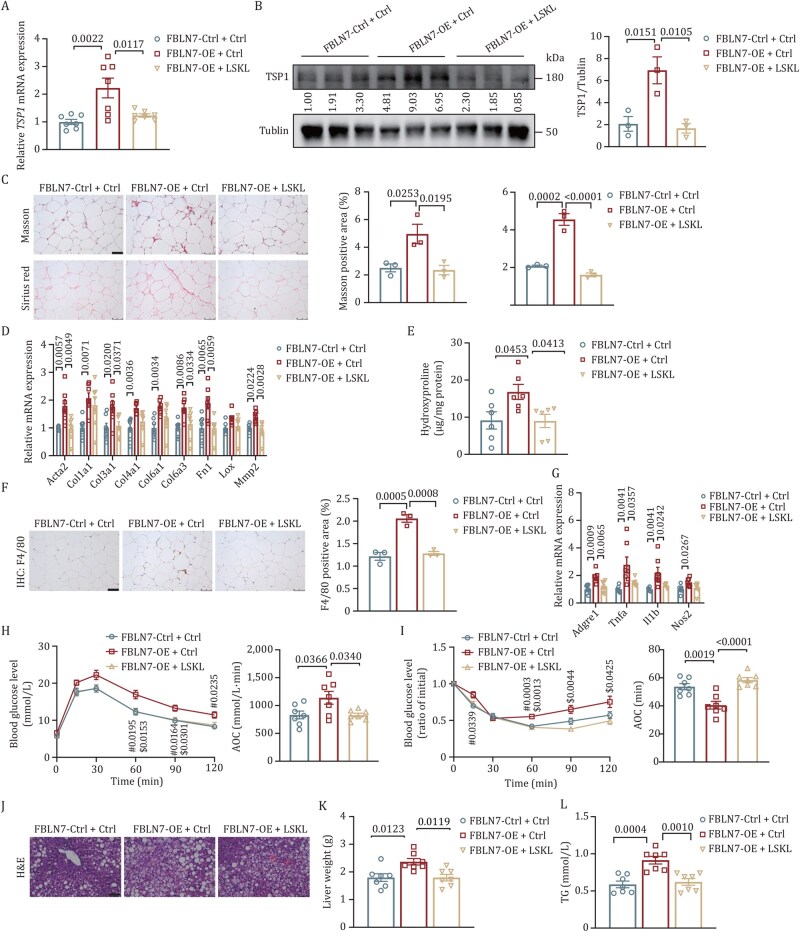
**FBLN7 mediates pro-fibrosis signaling via TSP1 *in vivo***. *FBLN7*-OE or control mice received intraperitoneal injections of LSKL peptide or control vehicle. (A) RT-qPCR indicating *TSP1* mRNA level in iWAT (*n *= 7). (B) Western blot and quantification of TSP1 in iWAT (*n *= 3). (C) Representative Masson’s trichrome and Sirius Red staining images and quantitative analysis of iWAT (*n *= 3). Scale bars, 75 µm. (D) RT-qPCR indicating the mRNA abundance of pro-fibrosis genes in iWAT (*n *= 7). (E) Hydroxyproline contents of iWAT (*n *= 6). (F) Representative F4/80 IHC staining and quantitative analysis of iWAT (*n *= 3). Scale bars, 75 µm. (G) RT-qPCR indicating the mRNA abundance of pro-inflammatory genes in iWAT (*n *= 7). (H) GTT and AOC (*n* = 7. (I) ITT and AOC (*n* = 7). (J) Representative images of H&E staining of the liver. (K) Liver weight (*n *= 7). (L) Serum TG levels (*n *= 7). Data are presented as mean ± SEM. One-way ANOVA with Tukey’s multiple comparison test was performed for (A–I, K, and L). Two-way ANOVA with Tukey’s multiple comparison test was performed for (H) and (I). (H and I) ^**#**^*P* values between *FBLN7*-Ctrl + Ctrl and *FBLN7*-OE + Ctrl mice. ^$^*P* values between *FBLN7*-OE + Ctrl and *FBLN7*-OE + LSKL mice.

To solidify our understanding of how FBLN7 regulates WAT fibrosis via TSP1 *in vivo*, we intraperitoneally injected either LSKL (1 mg/mL) or control vehicle in *FBLN7*-OE and control mice. These mice had already been on an HFD for 3 weeks, and the injections were given three times a week for a total of 24 weeks. Consistent with in vitro findings, the administration of LSKL was able to attenuate the upregulation of TSP1 expression that was induced by *FBLN7* overexpression (Fig. 7A and 7B). Besides, the delivery of LSKL reduced ECM accumulation in HFD-fed *FBLN7*-OE mice, as revealed by the results of Masson’s trichrome and Sirius red staining (Fig. 7C), along with decreased expression of pro-fibrosis genes (Fig. 7D) and lowered levels of hydroxyproline (Fig. 7E). In line with this, LSKL treatment also resulted in a decline in the expression of pro-inflammatory markers, as determined by F4/80 IHC and RT-qPCR analyses (Fig. 7F and 7G). Moreover, LSKL administration reversed the deteriorated glucose metabolic abnormalities and insulin resistance that were caused by *FBLN7* overexpression, as revealed by glucose and insulin tolerance tests (Fig. 7H and 7I). Additionally, LSKL treatment alleviated liver steatosis and decreased serum TG levels (Fig. 7J–L). These results suggest that the induction of AT fibrosis and subsequent metabolic deterioration by FBLN7 are, at least in part, dependent on TSP1-mediated regulation.

### FBLN7 binds to TSP1 and promotes TGF-β activation

**Figure 8.**
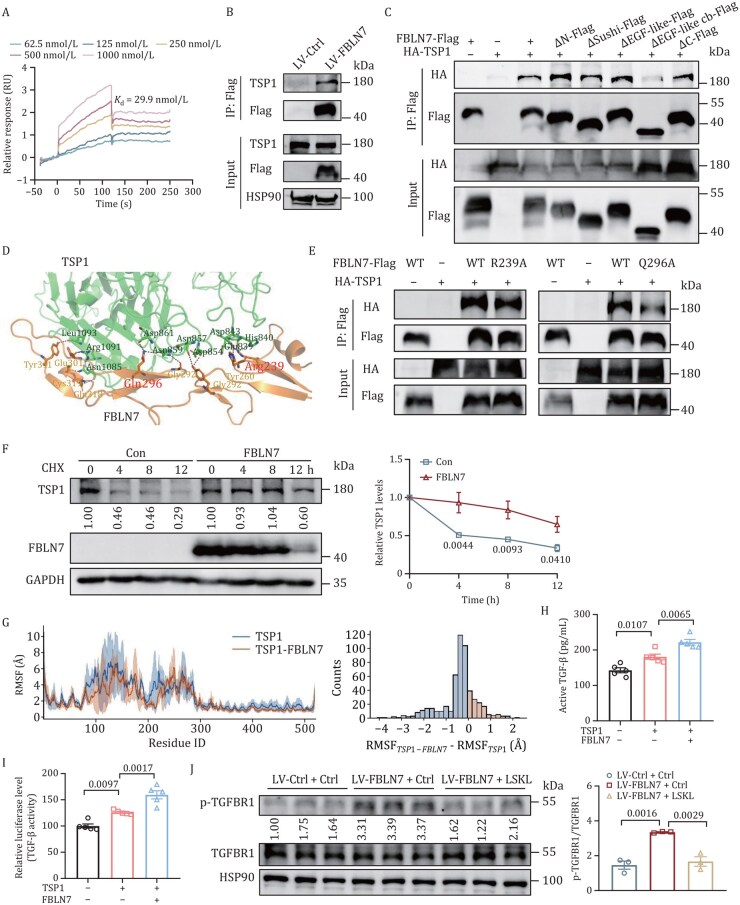
**FBLN7 interacts with TSP1 and promotes TGF-β activation**. (A) Biacore sensorgram depicting real-time binding kinetics of FBLN7 to immobilized TSP1. Sequential injections of FBLN7 induce concentration-dependent increases in response units. (B) IP of flag from SVF cells infected with Flag-empty control (LV-*Ctrl*) or Flag-FBLN7 (LV-*FBLN7*), followed by immunoblot analysis. (C) The HEK293T cells were co-transfected with plasmids encoding HA-tagged TSP1 and either full-length FBLN7-Flag or its deletion mutants, followed by Co-IP assay. (D) Predicted binding sites of FBLN7 to TSP1 via protein–protein docking. (E) Co-IP analysis of TSP1 interaction with FBLN7 mutants R239A and Q296A in HEK293T cells. (F) Representative Western blot and quantification of TSP1 in HEK293T cells treated with CHX for the indicated time (*n *= 3). The cells were transfected with HA-TSP1 alone or co-transfected with FBLN7 overexpression plasmid. (G) Root mean square fluctuation of TSP1 and TSP1-FBLN7 complex. (H) Bioactive TGF-β1 levels in HEK293T cell supernatant. The cells were transfected with the indicated plasmids and then treated with latent TGF-β1 for 24 h (*n *= 5). (I) The activity measurement of TGF-β using NIH-3T3 TGF-β signaling reporter cells (*n *= 5). CM from HEK293T cells transfected with the indicated plasmids was collected and applied to reporter cells in the presence of latent TGF-β (50 ng/mL). (J) Western blot and quantification of p-TGFBR1 and TGFBR1 in LV-*Ctrl* or LV-*FBLN7* cells after LSKL treatment (*n *= 3). Data are presented as mean ± SEM (H–J). Two-way ANOVA with Sidak’s multiple comparison tests was performed for (F). One-way ANOVA with Tukey’s multiple comparison tests was performed for (H–J).

To elucidate the mechanisms by which FBLN7 exerts its effects via TSP1, we delved into the molecular link between these two proteins. Given that FBLN7 and TSP1 are ECM proteins capable of interacting with other ECM components, we hypothesized that FBLN7 modulates TGF-β/Smad signaling through its interaction with TSP1. We initiated our investigation by performing Biacore analysis to examine the interaction between FBLN7 and TSP1 (Fig. 8A). Co-immunoprecipitation (Co-IP) analysis on HEK293T cells showed that TSP1 immunoprecipitated with FBLN7, and this interaction persisted when FBLN7 immunoprecipitating with TSP1 (Fig. S12G). Moreover, we confirmed this interaction in ASPCs (Fig. 8B). We then explored the key FBLN7 domain responsible for TSP1 binding. We constructed five plasmids, each lacking one of the primary functional domains of FBLN7: the coiled coil in N-terminus (ΔN, residues 22–79), the sushi domain (ΔSushi, residues 73–142), the EGF-like domain (ΔEGF-like, residues 136–172), the EGF-like calcium-binding domain (ΔEGF-like cb, residues 224–320), and the C-terminus (ΔC, residues 374–440) (Fig. S12H). Co-IP results revealed that FBLN7 lacking the EGF-like cb domain exhibited impaired interaction with TSP1, whereas full-length FBLN7, as well as ΔN, ΔSushi, ΔEGF-like, and ΔC bound to TSP1 with equal affinity (Fig. 8C), indicating that FBLN7 binds to TSP1 through its EGF-like cb domain. To further understand the molecular details underlying the interaction between the EGF-like cb domain of FBLN7 and TSP1, we performed protein–protein docking of these two proteins. The results showed that FBLN7 interacted with a disordered region (residue number 839-861) and β-sheets in the C-terminal of TSP1, forming a complex H-bond network. Further MM/GBSA4 calculations and energy decomposition analysis suggested that Arg239 and Gln296 on FBLN7 were dominant in the protein–protein interaction (PPI). Specifically, Arg239 contributed a binding free energy of −9.27 kcal/mol, and Gln296 contributed −4.07 kcal/mol (Fig. 8D). Through site-directed mutants of these two sites to alanine, we found that both mutants weakened the binding of FBLN7 to TSP1, suggesting that Arg239 and Gln296 are crucial for the interaction between these two proteins (Fig. 8E).

Interestingly, when HEK293T cells were treated with the protein synthesis inhibitor cycloheximide (CHX), overexpression of FBLN7 resulted in a notably extended half-life of the TSP1 protein (Fig. 8F), suggesting that FBLN7 enhances the stability of the TSP1 protein. Next, we conducted molecular dynamics (MD) simulations for both TSP1 and the TSP1-FBLN7 complex and calculated the root mean square fluctuation (RMSF) for the equilibrated trajectories. Our findings revealed that the flexibility of TSP1 residues decreased following the interaction with FBLN7 (Fig. 8G), indicating its improved thermal stabilization. Moreover, denatured IP experiments further confirmed that FBLN7 binding markedly decreased the K48 ubiquitination of TSP1 in HEK293T cells (Fig. S12I). These results may represent the underlying mechanism through which FBLN7 stabilizes the TSP1 protein structure.

Given that TSP1 is known to promote TGF-β/Smad signaling by activating latent TGF-β, we further found that in the presence of FBLN7, TSP1 triggered a significantly greater release of bioactive TGF-β from its latent form (Fig. 8H). In addition, by using NIH-3T3 cells that stably expressed a luciferase reporter vector containing four repeats of a Smad-binding element upstream of the minimal promoter of the firefly luciferase coding region (Fan et al., 2019), we confirmed that FBLN7 overexpression led to a substantial increase in the production bioactive TGF-β (Fig. 8I). Finally, in line with the notion that TSP1 could activate the latent TGF-β1 to evoke TGF-β signaling through phosphorylated TGFBR1, we found that FBLN7 overexpression indeed activated the phosphorylation of TGFBR1, whereas this effect was weakened when TSP1 was inhibited or knocked down (Figs. 8J and S12J). Altogether, these data indicate that FBLN7 interacts with TSP1, facilitating the conversion of latent TGF-β into its active form. This, in turn, enhances the TGFBR1/Smad signaling pathway and ultimately promotes AT fibrosis.

### FBLN7-neutralizing antibody alleviates obesity-related AT fibrosis and improves systemic metabolic homeostasis

**Figure 9.**
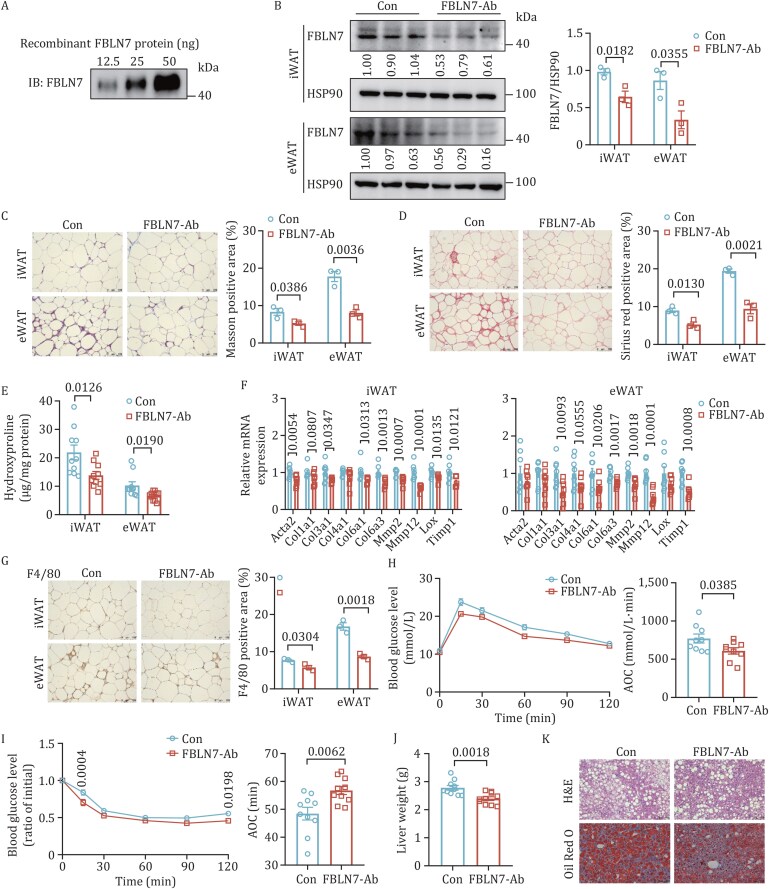
**FBLN7 neutralizing antibody alleviates obesity-related AT fibrosis and improves metabolic homeostasis**. *C57BL*/*6J* mice received intraperitoneal injections of anti-FBLN7 neutralizing antibody or IgG control. (A) Confirmation of anti-FBLN7 antibody binding by Western blot. (B) Western blot and quantification of FBLN7 in WAT (*n *= 3). Representative Masson’s trichrome (C) and Sirius Red staining images (D) and quantitative analysis (*n *= 3). Scale bars, 100 µm. (E) Hydroxyproline content in WAT (*n *= 10). (F) RT-qPCR indicating the mRNA abundance of pro-fibrosis genes in WAT (*n *= 8). (G) Representative F4/80 IHC staining and quantitative analysis (*n *= 3). Scale bars, 100 µm. (H) GTT and AOC (*n* = 9). (I) ITT and AOC (*n* = 10). (J) Liver weight (*n *= 10). (K) Representative images of liver H&E (top) and Oil Red O (bottom) staining (*n *= 3). Scale bars, 100 µm. Data are shown as mean ± SEM. Two-tailed Student’s *t*-test was performed for (B–J). Two-way ANOVA with Sidak’s multiple comparison test was performed for (H) and (I).

To further evaluate the potential therapeutic effects of FBLN7 inhibition *in vivo*, we developed a neutralizing antibody to interfere with FBLN7 and administered it to HFD-induced mice. In detail, 12-week HFD mice received intraperitoneal injections of either anti-FBLN7 neutralizing antibody (250 µg per mouse) or control IgG antibody three times per week for a duration of 8 weeks. The specificity and efficiency of the neutralizing antibody were confirmed by Western blot analysis (Fig. 9A and 9B). Additionally, we detected the protein levels of other members of the fibulin family. The expression levels of these fibulins remained unchanged in the antibody-treated group compared to the control group (Fig. S13A–E), further confirming the specificity of the neutralizing antibody. Compared with mice receiving IgG controls, mice treated with FBLN7-neutralizing antibody exhibited a marked reduction in ECM accumulation, as evidenced by Masson’s trichrome and Sirius red staining (Fig. 9C and 9D). In agreement, the hydroxyproline content, along with the expression levels of pro-fibrosis genes, were also significantly reduced (Fig. 9E and 9F). In addition, the abundance of F4/80-positive macrophages and genes involved in inflammation were notably decreased (Figs. 9G and S13F). The neutralizing FBLN7 treatment could also improve glucose tolerance and insulin sensitivity in HFD-fed mice (Fig. 9H and 9I). Furthermore, mice treated with FBLN7 neutralizing antibody had lower liver weights (Fig. 9J), reduced hepatic lipid accumulation (Fig. 9K), and decreased serum levels of TG, TC, and NEFA (Fig. S13G–I). Collectively, these results demonstrate that the FBLN7 neutralizing antibody can effectively prevent the progression of AT fibrosis and inflammation, while offering beneficial metabolic effects, indicating that blocking FBLN7 might be a promising therapeutic target for obesity-induced AT fibrosis and associated metabolic disorders.

## Discussion

The development of AT fibrosis in response to excess caloric intake is considered a maladaptive mechanism that disrupts AT homeostasis, which is critical for maintaining systemic metabolic health (Marcelin et al., 2022). Mature adipocytes, inflammatory cells, and fibroblasts all have effects on AT fibrosis (Marcelin et al., 2019). In addition, recent evidence has suggested that DPP4^+^PDGFRα^+^ adipocyte progenitors could also aggravate AT fibrosis by reducing the expression of fat identity genes and promoting ECM accumulation (Merrick et al., 2019). Given the heterogeneous nature of adipocyte progenitors, it becomes imperative to determine the specific subsets that actively participate in the development of AT fibrosis. In our research, we conducted scRNA-seq on the stromal vascular cells of AT and identified ASPC2 subpopulation of PDGFRα^+^ ASPCs. This subpopulation, exclusively and highly expressing ECM function-related genes, as well as *FBLN7* in response to HFD challenge, has emerged as a new driver of AT fibrosis in obesity.

In this study, we have identified FBLN7 in ASPCs as a novel regulator of AT fibrosis. Specifically, *FBLN7* deletion in ASPCs protects AT function against pathological remodeling, resulting in reduced fibro-inflammation and improved systemic metabolic homeostasis. Conversely, overexpression of FBLN7 yields the opposite effect. Notably, the improved systemic metabolic profiles observed in *FBLN7*-KO and *FBLN7*-APKO mouse models mainly resulted from the ameliorated fibro-inflammatory environment of AT, which ensured the safe storage of energy and prevented lipotoxicity in peripheral tissues. This is consistent with the established notion that the optimal health and functionality of WAT is pivotal in maintaining systemic metabolic homeostasis (Reyes-Farias et al., 2021). Hence, therapeutic interventions targeting the anti-fibrotic regulatory pathways of ASPCs hold promise for promoting healthy WAT remodeling and metabolic balance related to obesity.

Fibulins comprise eight ECM glycoproteins that influence various cellular processes, owing to their complex interactions with other ECM molecules and cellular receptors (Mahajan et al., 2021). As the newest discovered member of this family, FBLN7 remains relatively enigmatic, with limited knowledge of its functions. Recently, Zheng et al. demonstrated that *FBLN7* deficiency alleviated the pathological cardiac remodeling triggered by myocardial infarction (Zheng et al., 2023). Our findings further establish the role of FBLN7 as a matricellular protein in the development of tissue fibrosis. Additionally, consistent with previous reports highlighting the regulatory influence of fibulins on TGF-β signaling pathways (Lee et al., 2008; Liu et al., 2016; Tian et al., 2015; Tsuda et al., 2018), here we also reveal that the stimulatory impact of TGF-β on fibrogenic responses and collagen deposition is diminished in *FBLN7*-KO ASPCs, whereas overexpressing *FBLN7* increases the response to the pro-fibrotic effects of TGF-β signaling.

We propose that the interaction between FBLN7 and TSP1 represents the primary molecular mechanism by which FBLN7 amplifies TGF-β/Smad signaling. TSP1 has been reported to bind to the TGF-β precursor and facilitate its conversion into a biologically active form in a protease-independent manner, whether in solution, at the cell surface, or in the extracellular milieu, thereby initiating the TGF-β/Smad pathway (Gu et al., 2022; Sweetwyne and Murphy-Ullrich et al., 2012). Our findings indicate that the FBLN7-TSP1 complex cooperates with TGF-β to drive fibrotic processes, and that inhibition of TSP1 function or its KD represses the fibrotic-promoting effects of FBLN7 both *in vitro* and *in vivo*. Interestingly, our results reveal the existence of a pro-fibrotic positive feedback loop, wherein the FBLN7-TSP1 complex phosphorylates Smad3, enabling p-Smad3 to translocate to the nucleus and further upregulate *TSP1* transcription; TSP1, in turn, binds latent TGF-β1, activating TGF-β/Smad signaling and perpetuating this pro-fibrotic signaling loop. Therefore, it is reasonable that FBLN7 deficiency could lead to the downregulated of expression of TSP1. In addition, prior studies have indicated that the EGF-like domain facilitates interactions between FBLN1 and ECM proteins, such as fibronectin and amyloid precursor protein (Ohsawa et al., 2001; Tran et al., 1997). In line with this, we reveal that the EGF-like cb domain is the specific region responsible for the binding of FBLN7 to TSP1.

In the present study, we used HFD-induced global and conditional KO mouse models to investigate the influence of FBLN7 on AT fibrosis in obesity. Nevertheless, AT fibrosis is multifaceted and can be attributed to other mechanisms. Notably, other pathological conditions that compromise systemic metabolic health, such as aging (Tchkonia et al., 2010), can also accelerate the progression of AT fibrosis. Recently, a distinct subpopulation of PDGFRα^+^ ASPCs has been characterized as aging-dependent regulatory cells (ARCs) (Nguyen et al., 2021). Our findings further uncover that *FBLN7* mRNA levels in WAT exhibit an age-dependent increase (data not shown); therefore, the role and underlying mechanisms by which FBLN7 in ASPCs function in age-related AT fibrosis warrant further investigation. Moreover, compared to *C57BL*/*6J* background mice, *C3H*/*HeOuj* (*C3H*) mice are more prone to develop AT fibrosis (Marcelin et al., 2017), and thus employing *C3H* mice could yield additional evidence to elucidate the relationship between FBLN7 and AT fibrosis. Lastly, given that *PDGFRα*-Cre is also expressed in various other cell types of neuroectodermal or mesenchymal origin, including oligodendrocytes (Rivers et al., 2008) and neural crest cells (Soriano, 1997), it is crucial to further ascertain whether the reduced fibro-inflammation and improved metabolic health observed in *FBLN7*-APKO mice are attributed not solely to AT.

Our mouse studies revealed that ASPC-derived FBLN7 modulated local adipose fibrosis and systemic metabolic dysfunction, a finding corroborated in human visceral fat. However, several key questions remain to be addressed: (1) whether FBLN7 can be secreted into circulation and serve as a biomarker for adipose fibrosis and metabolic diseases, and (2) to what extent adipose precursor cells contribute to circulating FBLN7 levels. Developing FBLN7-specific ELISA assays would be essential for addressing these questions in future investigations. Furthermore, we have identified a human FBLN7 missense variant V99E, which is linked with enhanced insulin sensitivity. Nevertheless, the mechanism by which the V99E affects the function of FBLN7 and regulates metabolic homeostasis requires further investigation.

## Supplementary Material

pwaf084_Supplementary_Materials

## Data Availability

The raw sequence data reported in the present study are available in the Gene Expression Omnibus (GEO) with accession numbers GSE308197 (scRNA-Seq) and GSE308198 (RNA-seq).

## Code availability

Codes are available from the corresponding authors upon reasonable request.
