# Supplementary material for: Fibulin-7 in progenitor cells promotes adipose tissue fibrosis and disrupts metabolic homeostasis in obesity
Source: Protein Cell. 2025 Oct 23;17(3):212–30. doi: 10.1093/procel/pwaf084 (PMC13064845; doi:10.1093/procel/pwaf084)
Supplement: pwaf084_Supplementary_Materials [file pwaf084_supplementary_materials.pdf]

Materials and Methods

Human studies

Cohort 1. Participants were classified as either having a normal weight (BMI < 24) or being overweight and obese (BMI ≥ 24) according to the Working Group on Obesity in China criteria. Visceral fat was obtained from age- and sex-matched human donors undergoing scheduled routine surgery. The tissue biopsies were freed from visible connective tissue, rinsed to remove blood and clots, immediately frozen, and stored in liquid nitrogen until further processing.

Cohort 2. A total of 11,015 subjects were included for human *FBLN7* genotyping analysis from Shanghai Nicheng cohort. Genotyping was performed by Infinium Asian Screening Array and Multi-Ethnic Genotyping Array. The statistical analysis was performed by PLINK.

Animal experiments

Animal breeding

The animals received humane care according to the Guide for the Care and Use of Laboratory Animals published by the National Academy of Sciences and National Institutes of Health. All mice were fed a standard chow diet (P1200F, Shanghai Puluteng) or an HFD (60% kcal fat, D12492, Research Diets). The mice were monitored daily and weighed weekly. At the end of the experiment, the mice were euthanized and the tissues were harvested.

C57BL/6J mice were purchased from GemPharmatech. The *FBLN7*-KO mice were

commissioned by Cyagen Biosciences. The CRISPR/Cas9 strategy was used to delete exon 2-3 of *FBLN7* via nonhomologous recombination. *FBLN7*-Flox mice were generated by GemPharmatech, in which exon 2-3 of the *FBLN7* allele was flanked by loxP sites. *FBLN7*-APKO mice were generated by mating *FBLN7*-Flox mice with *PDGFRα*-Cre mice (013148, Jackson Laboratory). The mice were genotyped by PCR using DNA isolated from their tails. The primers used for genotyping are listed in **Table S3**.

For the local iWAT overexpression model, we generated an AAV9-mediated overexpression of *FBLN7* or a negative control driven by the CMV promoter (GeneChem). *C57BL/6J* mice were anesthetized under isoflurane (5% induction, 2% maintenance), and AAV was directly injected into the iWAT on both sides (5×10^{10} vg). To overexpress *FBLN7* specifically in PDGFRα^+ progenitor cells, we used Cre-driven AAV8-mediated overexpression of *FBLN*7 (GeneChem). The AAV-DIO-*FBLN7* or AAV-DIO-*Ctrl* were injected into the iWAT of *PDGFRα*-Cre mice.

Metabolic phenotyping

For glucose tolerance tests (GTT), the mice were intraperitoneally injected with glucose (1.0-2.0 g/kg). For insulin tolerance tests (ITT), insulin was administered intraperitoneally (1.5-2.5 U/kg). Blood glucose levels were measured via the tail vein immediately before injection and at several time points after injection. The area over the curve (AOC) was determined. For the acute insulin challenge, mice received an intraperitoneal injection of insulin (2 U/kg body weight) after 4 h of fasting. Tissues were harvested 15 min after injection.

For hepatic triglyceride, liver tissues (30-50 mg) were homogenized in PBS and mixed with a chloroform: methanol solution (2:1[v/v]). The organic phase was then transferred, air-dried overnight, and re-suspended in absolute ethanol containing 1% TritonX-100. Triglyceride concentrations were determined using an automatic biochemical analyzer. Insulin levels were measured using Ultra Sensitive Mouse Insulin ELISA Kit (90080, Crystal Chem). Serum adiponectin levels were detected using ELISA kits (MRP300, R&D Systems). Serum TG, TC, and NEFA levels were assayed using an automatic biochemical analyzer.

Collagen content

Hydroxyproline levels were measured using a hydroxyproline colorimetric assay. In brief, frozen adipose tissues were weighed, heated in 6 N HCl at 120°C for 3 h in sealed tubes, and then dried at 60°C for 4 h. After dissolution in isopropanol, the solution was transferred to a 96-well plate. Chloramine-T was used to oxidize free hydroxyproline to produce pyrrole; p-dimethylamino-benzaldehyde (DMAB) was subsequently added for colorimetric quantification. The absorbance was read at 560 nm, and the concentration was determined using a hydroxyproline standard curve.

Histological analysis

Adipose tissue and liver were fixed with 4% paraformaldehyde, embedded in paraffin, sectioned into 5-μm-thick sections, and processed for Sirius red, Masson's trichrome and H&E staining. Frozen sections were prepared for Oil Red O staining. For IHC, the sections were incubated with primary antibodies against F4/80 (14-4810-82, eBioscience, 1:50) and FBLN7 (ab122240, Abcam, 1:50), followed by incubation with

secondary antibodies conjugated with horseradish peroxidase. Quantification was performed using ImageJ (National Institutes of Health). The theoretical number of adipocytes was determined by calculating the ratio of WAT weight to the average cell volume (Hsiao et al., 2020).

Flow cytometry test

Freshly isolated SVF cells from WAT were re-suspended in staining buffer (BD Pharmingen) and stained with indicated fluorescent-conjugated antibodies for 30 min at 4°C in the dark. The antibodies used were anti-CD45-PE-Cy7 (552848, BD Biosciences), anti-PDGFRα-PE (12-1401-81, eBioscience), and anti-Sma-AF488 (53-9760-82, eBioscience), and anti-CD45-FITC (109805, Biolegend). The corresponding antibodies for isotype controls were PE-Cy7 Rat IgG2b kappa (552849, BD Biosciences), PE Rat IgG2a kappa (12-4321-80, eBioscience), AF488 Mouse IgG2a kappa (53-4724-80, eBioscience), and FITC Mouse IgG2a kappa (400207, Biolegend), respectively. DAPI (C1002, Beyotime), fixable viability stain (FVS) 780 (565388, BD Biosciences), and FVS 510 (564406, BD Biosciences) were used to differentiate live and dead cells. The samples were subjected to flow cytometry analysis using an ImageSteam Mark II Imaging Flow Cytometer (Luminex). Data were analyzed using the IDEAS software (Version 6.2).

FBLN7 neutralizing antibody.

The FBLN7 neutralizing antibody was developed by our group and MabStar Antibody Technology, according to the standard hybridoma technique (Alejandra et al., 2023). In brief, hybridoma cells secreted mouse monoclonal antibodies (mAbs) against mouse

FBLN7 peptide 271-281aa (DVDECAGPQHM). The mouse mAbs were purified by Protein A affinity chromatograph. The BCA protein assay kit was used to measure the concentration of the obtained antibody, according to the manufacturer's instructions. The purified antibody was diluted in saline and then injected into mice.

Cell culture and stimulation

The WAT were isolated, minced, and digested in collagenase II (C6885, Sigma). The digest was centrifuged, and the floating adipocytes were subsequently discarded. The SVF cells were filtered through a 40 μm cell strainer, centrifuged, and then re-suspended and cultured in DMEM (11995, GIBCO) containing 10% fetal bovine serum (FBS) (10099, GIBCO) and 1% penicillin/streptomycin (15140-122, GIBCO).

For mimicking a pro-fibrotic environment, cells were stimulated with human recombinant TGF-β1 (100-21C, Peprotech, 5 ng/mL for eWAT and 10 ng/mL for iWAT). To inhibit TSP1 function, cells were treated with LSKL (10 μM, HY-P0299, MedChemExpress). Cells were stimulated with TGF-β1 or LSKL without FBS in DMEM for 6 h to detect p-Smad2/3 levels and for 24 h to measure the mRNA levels of pro-fibrosis genes and α-SMA.

HEK293T cells (GNHu17, Cell Bank of Type Culture Collection of the Chinese Academy of Sciences, Shanghai, China) were transfected with the indicated plasmids using Lipofectamine 2000 (116668019, Invitrogen) and then subjected to further tests. The cells were treated with CHX (100 μg/mL HY-12320, MedChemExpress) for protein stability experiments.

The adipocyte differentiation tests were performed as previously described (Yan et al., 2022). The differentiated adipocytes were treated with PA (200 μM, P5585, Sigma). The conditioned medium of adipocytes was collected after 24 h and then used to treat progenitor cells. For Oil Red O staining, cells were fixed with 4% paraformaldehyde for 20 min, followed by Oil Red O (00625, Sigma) incubation for 30 min.

For CCK8 assay, progenitor cells were seeded into a 96-well plate. CCK8 reagent (Beyotime, C0041) was added to each well and optical density value was detected using a microplate reader at 450 nm. For EdU assay, cells were cultured in 24-well plates, incubated with 10 μM EdU for 2 h, and then washed, fixed, neutralized, and permeabilized according to manufacturer's protocol (Beyotime, C0071). The nuclei were stained with Hoechst for 10 min.

Virus-mediated gene transfer

SVF cells were prepared and infected at a multiplicity of infection (MOI) of 50-100 with lentivirus-shRNA (sh-*FBLN7* or sh-*TSP1*), or lentivirus overexpressing *FBLN7*. All the viruses were prepared by GeneChem. The cells were infected with lentivirus for 48 h before further treatment.

Plasmid construction

Full-length cDNA of mouse *TSP1* and *FBLN7* were amplified by standard PCR and sub-cloned into the pcDNA 3.1 vector with a C-terminal HA-, Flag-, or Myc- tag. pcDNA 3.1-HA, pcDNA 3.1-Flag, and pcDNA 3.1-Myc plasmids were used as controls.

FBLN7 deletion mutants, p.R293A, and p.Q296A mutants of plasmids were constructed by BioVision Technology.

Immunofluorescence staining

The cells were seeded on glass coverslips, fixed for 10 min with 4% paraformaldehyde at room temperature, and washed with PBS. The cells were permeabilized for 10 min with 0.1% TritonX-100, and then incubated with blocking buffer (PBS containing 5% BSA) for 1 h at room temperature. Cells were stained with antibodies α-SMA (A2547, Sigma-Aldrich,1:1000) overnight at 4°C. After washing, the cells were incubated for 1 h with Cy3-conjugated goat anti-mouse secondary antibody (A0521, Beyotime, 1:1000). DAPI was used to label nuclei. Images were captured using a fluorescence microscope (Leica) and analyzed using the ImageJ.

Immunoprecipitation

The cells were lysed in IP lysis buffer (P10013, Beyotime) for 1 h at 4°C. After centrifugation, part of the supernatant was mixed with 5× loading buffer as the input sample. The remaining lysates were incubated with Agarose Conjugated Anti-DYKDDDDK Affinity beads (SA042005, Smart Life sciences) at 4°C overnight. Then the beads were washed thrice with IP lysis buffer and boiled with 2× loading buffer for 10 min to obtain the IP sample, followed by Western blotting using the indicated antibodies.

Measurement of bio-active TGF-β1

The NIH-3T3 reporter cells were kindly provided by Dr. Bing Sun (University of Chinese Academy of Sciences) and Dr. Yu Hu (Southern Medical University). The cells were treated with supernatant from control or treated HEK293T cells together with latent TGF-β1 (50 ng/mL, HY-P78360, MedChemExpress). After 24 hours of incubation, the cells were lysed and luciferase activity was assessed by light production from a luciferin substrate (E1910, Promega) using a luminometer. Alternatively, the bio-active TGF-β1 levels were measured using ELISA kits (DB100C, R&D Systems) as per manufacturer's instructions.

Affinity measurement

Surface Plasmon Resonance (SPR) measurements were performed using a BIAcore 8 K instrument (GE Healthcare) to measure binding affinities. Recombinant TSP1 protein (7859-TH-050, R&D) was diluted in a sodium acetate solution (pH 4.0) at a final concentration of 4 μg/mL and was immobilized on a CM5 sensor chip (GE Healthcare) to capture the recombinant FBLN7 protein (8606-FB-050, R&D). The FBLN7 protein (62.5, 125, 250, 500, and 1000 μM) was injected at a rate of 30 μL/min for 120 s in single-cycle mode. In the dissociation phase, the running buffer was injected at a rate of 30 μL/min for 120 s. The binding kinetics data were analyzed using the Biacore T200 Insight Evaluation Software.

Protein-Protein Docking of FBLN7 and TSP1

The 3D structures of FBLN7 (UniProt ID: Q501P1) and TSP1 (UniProt ID: P35441) were fetched from AlphaFold Database (Varadi et al., 2022). The EGF-like cb domain of FBLN7 (residue number 224-320) and the last 1000 residues of TSP1 (residue number 171-1170) were used for protein-protein docking. The docking was performed using the HawkDock server. TSP1 was input as the receptor while FBLN7 was input as the ligand, while other settings are by default. The top 1 result was adapted as the predicted binding pose. To further reveal main contributors in PPIs, the binding free energy in the top 1 pose was calculated using the MM/GBSA scheme followed by energy decomposition to residues on the HawkDock server. Residues with a significant decomposed energy were recognized important in PPIs. The visualization of H-bonds of PPIs was performed using Pymol 2.5.2.

Western blotting

Cell or tissue lysates were homogenized in RIPA buffer (P0013; Beyotime) containing protease (4693132001, Roche) and phosphatase inhibitors (4906837001, Roche). Protein concentrations were determined using the BCA protein assay kit (P00125, Beyotime). Equivalent amounts of total protein were separated using sodium dodecyl sulfate-polyacrylamide gel electrophoresis and transferred to nitrocellulose membranes (Millipore). After blocking and overnight incubation with primary antibodies, blots were incubated with horseradish peroxidase (HRP)-conjugated anti-mouse and anti-rabbit secondary antibodies. Images were captured using a Bio-Rad imager and

analyzed using ImageJ. The following antibodies were used: β-ACTIN (4970, 1:2000), phospho-AKT (4058, 1:1000), AKT (9272, 1:1000), Smad2/3 (8685, 1:1000), phospho-Smad2/3 (8828, 1:1000), TSP1 (37879, 1:1000), DYKDDDDK Tag (14793, 1:1000), HA-Tag (3724, 1:1000) and Myc-Tag (2278, 1:1000) obtained from Cell Signaling; GAPDH (sc-32233, 1:2000) and HSP90 (sc-13119, 1:2000), FBLN1 (sc-374539, 1:1000), FBLN3 (sc-33722, 1:1000) antibodies obtained from Santa Cruz Biotechnology; FBLN7 (1:1000) purchased from Proteintech; α-SMA (A2547,1:1000) and α-Tublin (T6199, 1:2000) from Sigma-Aldrich; Col1a1 (ab34710, 1:1000), FBLN2 (ab96138, 1:1000), FBLN4 (ab125073, 1:1000), and FBLN5 (ab66339, 1:1000) from Abcam; Col3a1 (NB600-594, 1:1000) from Novus Biologicals; TGFBR1 (A16983, 1:1000) from ABclonal and phospho-TGFBR1 (PA5-40298, 1:1000) from Thermo Fisher Scientific.

Quantitative RT-qPCR

Total RNA from tissues was extracted using QIAzol reagent, and total RNA from cells was extracted using TRIzol reagent. Total RNA (1 μg) was transcribed into cDNA using an RT Reagent Kit (TaKaRa). The RT-qPCR analyses were performed using SYBR Green Master Mix (Applied Biosystems) or a TaqMan fluorescence probe (Applied Biosystems, Thermo Fisher Scientific) using a QuantStudio Real-Time PCR System (Applied Biosystems). The relative mRNA levels were calculated using the comparative threshold cycle (CT) method. Primers used are listed in **Tables S4 and S5**.

RNA-seq and bioinformatics analysis

iWAT SVF cells were infected with LV-*FBLN7* or control and then stimulated with TGF-β1. Total RNA was extracted using TRIzol reagent. RNA libraries were constructed using VAHTS® Universal V6 RNA-seq Library Prep Kit for IlluminaVazyme (Cat.N401-02), and paired-end reads were obtained on the Novaseq 6000 platform. The quality of the RNA-seq data was estimated using RSeQC (version 2.6.4). DEGs were identified using the RankProd49 package in R based on FDR < 0.05.

scRNA-seq and data processing

SVF cells were isolated from a pool of two mice after 16 weeks of NCD or HFD. Single-cell capture and cDNA library preparation were performed using the Chromium Single Cell 39 Reagent Kit v2 (10× Genomics). Libraries were sequenced using an Illumina NovaSeq 6000 instrument. FASTQ files of the scRNA-seq reads were aligned to the mouse reference genome (GRcm38) to generate gene-barcode matrices using the 10× Genomics Cell Ranger Pipeline (version 7.2.0) with default parameters. The filtered gene expression matrices containing only the detected cell-associated barcodes were then imported into the Seurat (v4.3.0.1) R toolkit for further analysis and visualization. We first removed potential doublets and cells with a mitochondrial gene content higher than 5%. Multiple gene-barcode matrices from the samples were merged using Seurat and processed using the NormalizeData function with default parameters. The FindVariableFeatures function was used to identify the top 2,000 variable genes. Normalized data for the variable genes were scaled and centered using the ScaleData

function. Principal component analysis (PCA) was performed on the scaled and centered data using the RunPCA function to compute 50 PCs. Based on the PCA dimensions, we used the RunHarmony function in the harmony (v0.1.1) R package to integrate multiple samples and reduce the batch effect. Subsequently, the top 30 dimensions generated by harmony were used for clustering and Uniform manifold approximation and projection (UMAP) analysis using FindNeighbors and RunUAMP functions, respectively.

To determine clustering and cell type annotation, we employed the FindClusters function with a resolution of 0.8 to generate clusters and identified the gene expression markers for each cluster using the FindAllMarkers function with the Wilcoxon rank sum test. Clusters were annotated based on marker gene expression and biological pathways of top 30 markers. Those assigned to the same cell type were merged. Otherwise, clusters with high expression of multiple types of cell markers may be doublets and were removed. We used the FindMarkers function of Seurat with the Wilcoxon rank-sum test to identify DEGs between groups. The average levels of DEGs were calculated using the AverageExpression function in R. ASPCs were further subset to re-cluster to smaller sub-clusters with the same clustering workflow.

For GO and KEGG enrichment, analyses of pathways in top markers or DEGs were performed using R software package clusterProfiler (version 4.10.0). For each cluster, markers with the top 30 avg_log2FC rankings were selected and evaluated for enrichment in GO biological pathways or KEGG pathways. And DEGs were filtered to -0.9 < avg_log2FC < 0.9 and a Benjamini-Hochberg adjusted P-value < 0.01 for ASPC2

We performed trajectory analysis using monocle2 (Qiu et al., 2017) for ASPCs according to the general pipeline (https://cole-trapnell-lab.github.io/monocle-release/docs/), which involved in selecting, sorting, and filtering genes, estimating size factors, and subsequently reducing dimensionality using the DDRTree algorithm. Cellular state plots and cell type maps were used to visualize trajectories.

We conducted GSVA to assess variation of pathway activity of each ASPC subset via R software package GSVA. R package msigdbr was used to obtain gene set of Mus musculus with KEGG subcategory.

Statistics

All data are presented as means with SEM, median (interquartile range), and n (%). Quantitative traits with skewed distributions were logarithmically transformed to approximate univariate normality. Comparisons between two groups were performed using an unpaired two-tailed Student's t-test. Comparisons between more than two groups were performed using one-way ANOVA, followed by Tukey's multiple comparison tests. Comparisons between more than two groups and factors were conducted using two-way ANOVA, followed by appropriate post-hoc testing. Pearson's correlation analysis was used to estimate the correlation between data series. Data were analyzed using GraphPad Prism (version 8.3.0; GraphPad Software) or SAS software (version 8.0; SAS Institute). The statistical parameters and the number of replicates used per experiment are shown in the figure legends.

References

Alejandra WP, et al. Production of monoclonal antibodies for therapeutic purposes: A review. Int Immunopharmacol. 120, 110376 (2023).

Hsiao WY, et al. The lipid handling capacity of subcutaneous fat is programmed by mTORC2 during Development. Cell Rep. 33, 108223 (2020).

Qiu X, et al. Single-cell mRNA quantification and differential analysis with Census. Nat Methods. 14, 309-315 (2017).

Varadi M, et al. AlphaFold protein structure database: massively expanding the structural coverage of protein-sequence space with high-accuracy models. Nucleic Acids Res. 50, D439-D444 (2022).

Yan J, et al. GPSM1 impairs metabolic homeostasis by controlling a pro-inflammatory pathway in macrophages. Nat Commun. 13, 7260 (2022).

Supplemental Figures

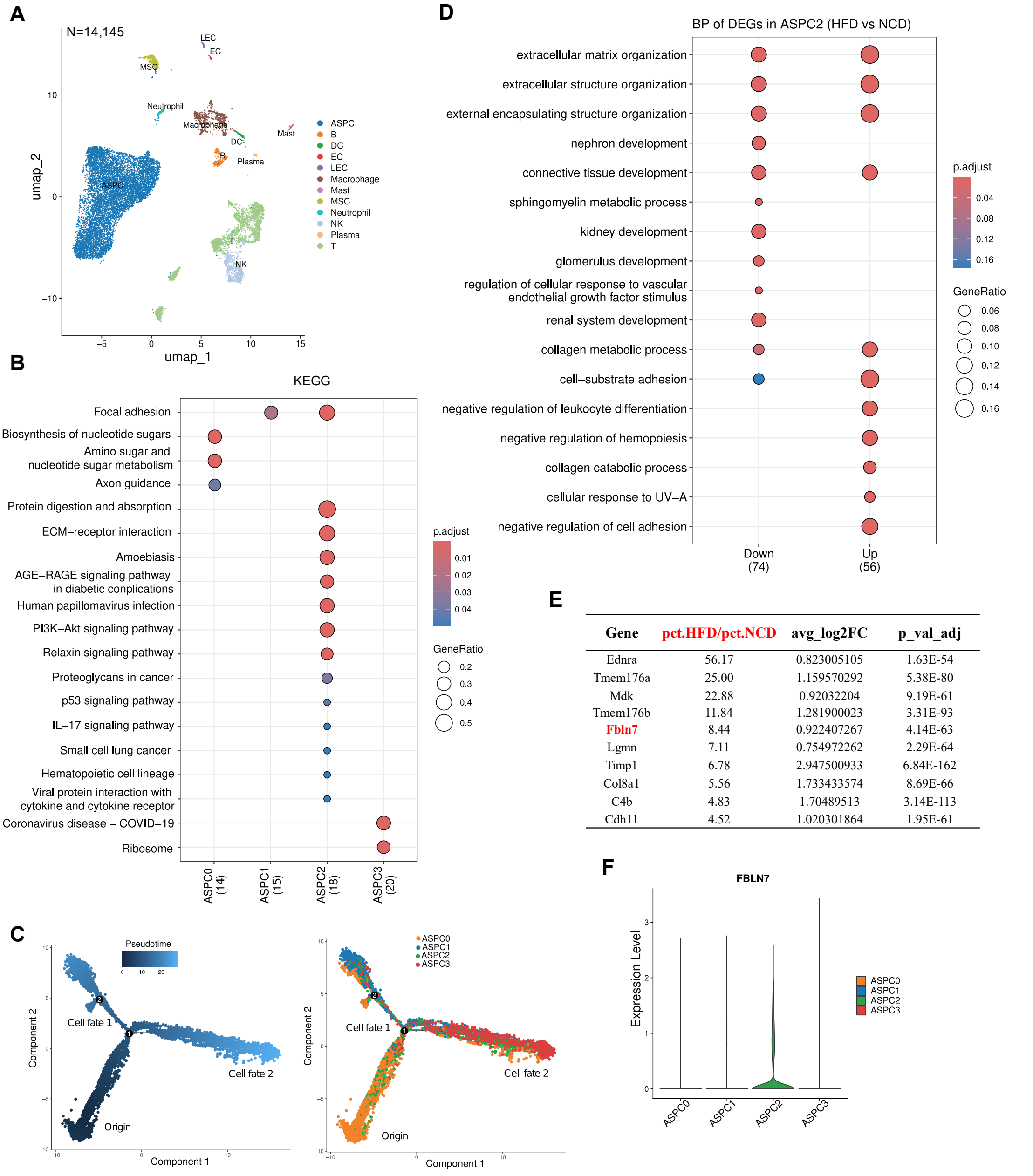

Gene	pct.HFD/pct.NCD	avg_log2FC	p_val_adj
Ednra	56.17	0.823005105	1.63E-54
Tmem176a	25.00	1.159570292	5.38E-80
Mdk	22.88	0.92032204	9.19E-61
Tmem176b	11.84	1.281900023	3.31E-93
Fbln7	8.44	0.922407267	4.14E-63
Lgmn	7.11	0.754972262	2.29E-64
Timp1	6.78	2.947500933	6.84E-162
Col8a1	5.56	1.733433574	8.69E-66
C4b	4.83	1.70489513	3.14E-113
Cdh11	4.52	1.020301864	1.95E-61

Supplemental Figure 1. ScRNA-seq reveals *FBLN7* upregulation in PDGFRα$^+$ ASPCs of murine AT in obesity

(**A**) UMAP plot of unsupervised clustering of 14,145 cells from the merged eWAT SVF of NCD and HFD mice. (**B**) KEGG pathway analysis of ASPCs clusters using top 30 marker genes. (**C**) Pseudotime trajectory analysis of ASPC clusters. Cells are colored by development states (left) and cluster identity (right). (**D**) GO analysis of ASPC2

using DEGs between NCD and HFD. (**E**) Top 10 genes ranked by the ratio of expression prevalence (percentage of gene-expressing cells) in HFD versus NCD. (**F**) Violin plots showing *FBLN7* expression in ASPC clusters.

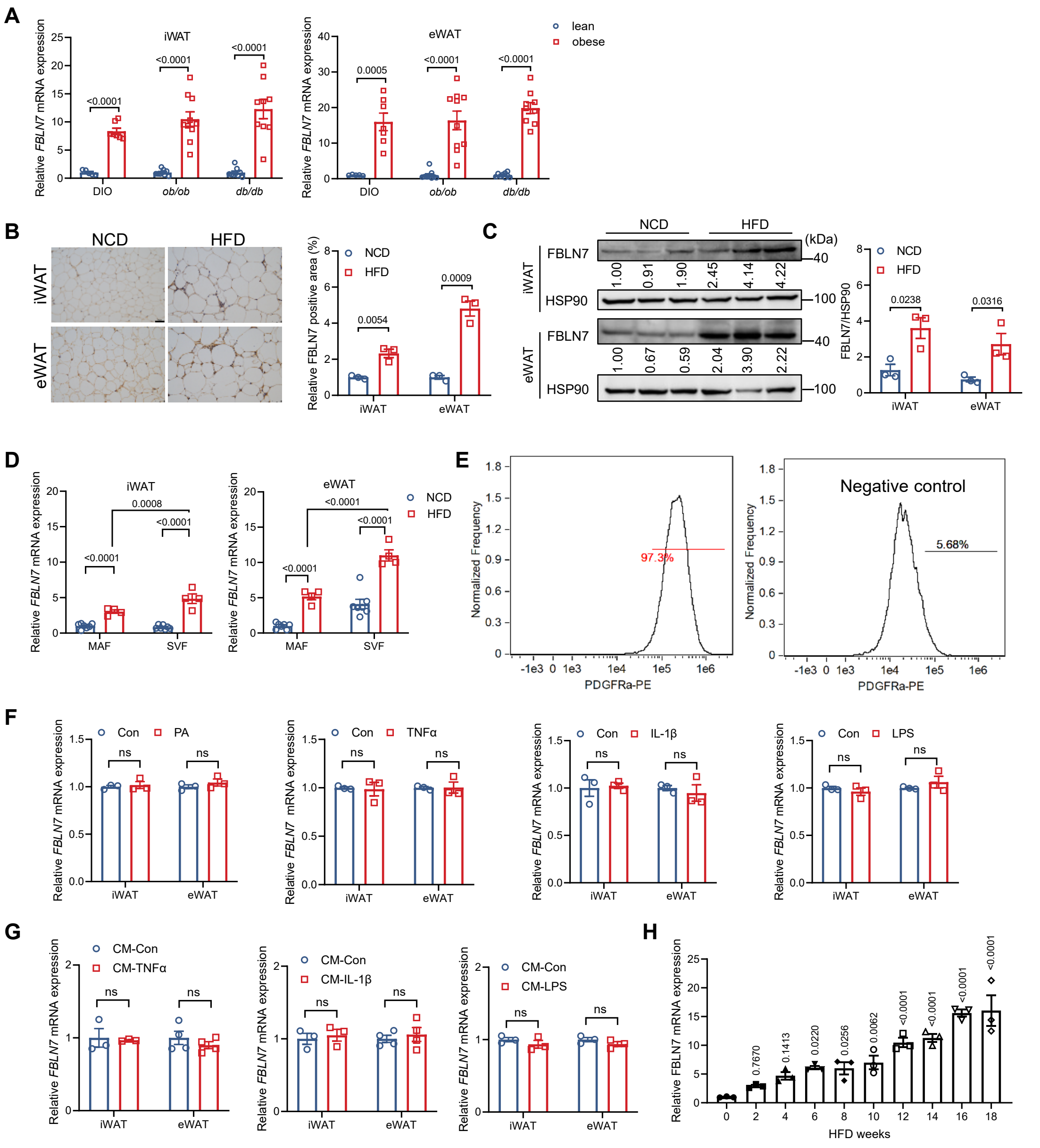

Supplemental Figure 2. *FBLN7* is upregulated in PDGFRα^{+} progenitor cells of murine AT in obesity

(**A**) RT-qPCR of *FBLN7* mRNA expression in WAT from lean and obese mice. DIO was established by feeding mice with NCD (n = 5) or HFD (n = 7) for 16 weeks. Genetic obesity models included: WT (n = 9) and *ob/ob* (n = 10) mice, and a separate group of WT and *db/db* mice (n = 9/group). (**B**) Representative IHC staining and quantitative analysis of FBLN7 in WAT from NCD and HFD mice (n = 3). Scale bars, 50 μm. (**C**) Western blot and quantification of FBLN7 in WAT from NCD and HFD mice (n = 3). (**D**) RT-qPCR of *FBLN7* mRNA expression in SVF and MAF isolated from WAT of NCD (n = 7) and HFD (n = 4) mice. (**E**) Flow cytometry analysis of PDGFRα^{+} in

adherent SVF cells. (**F**) RT-qPCR indicating *FBLN7* mRNA expression in progenitor cells of WAT treated with PA, TNFα, IL-1β, or LPS (n = 3). (**G**) CM was harvested from differentiated white adipocytes following treatment with PA, TNFα, IL-1β, or LPS. This adipocyte-derived CM was subsequently used to treat progenitor cells. RT-qPCR indicating *FBLN7* mRNA expression in progenitor cells (n = 3-4). (**H**) RT-qPCR indicating *FBLN7* mRNA expression in iWAT during HFD-induced obesity (n = 3)**.** Data are shown as mean ± SEM. Unpaired two-tailed Student's t-test was performed for **A-C, F,** and **G.** Two-way ANOVA was performed for **D**. For **H**, *P* value was determined by one-way ANOVA, with comparisons made against week 0.

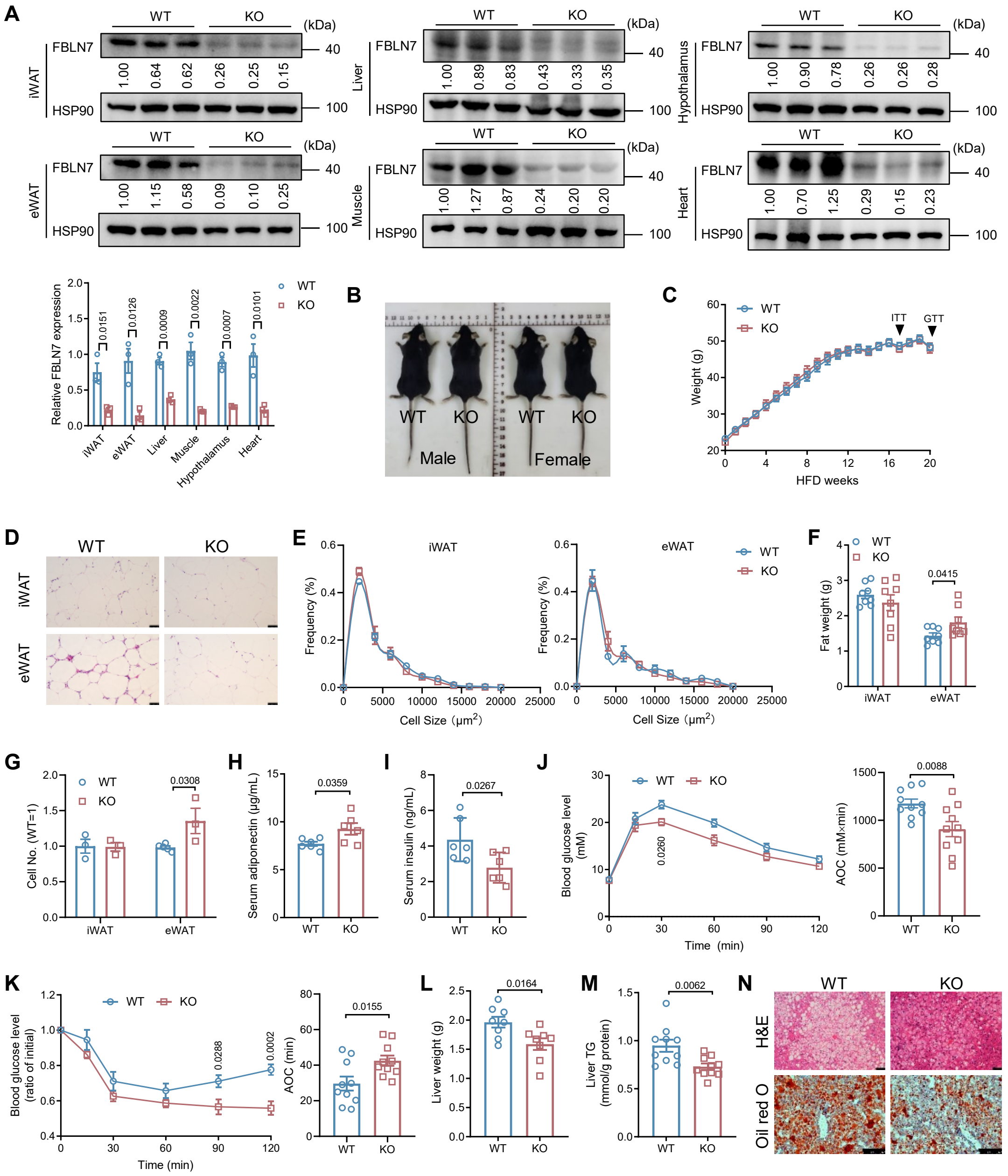

Supplemental Figure 3. Global FBLN7 ablation alleviates obesity-induced metabolic dysfunction

Data were compared between WT and KO mice. (**A**) Western blot and quantification of FBLN7 in different tissues (*n* = 3). (**B**) Representative image of 8-week-old mice. (**C**) Body weights during 20 weeks of HFD (*n* = 10). (**D**) Representative H&E images of WAT. Scale bars, 50 μm. (**E**) Quantification of adipocyte size (*n* = 3). (**F**) Fat pad weights (*n* = 8). (**G**) Quantification of adipocyte cell numbers (*n* = 3-5). (**H**) Serum adiponectin levels (*n* = 6). (**I**) Serum insulin levels (*n* = 6). (**J**) GTT and AOC (*n* = 10).

(**K**) ITT and AOC (*n* = 10). (**L**) Liver weight (*n* = 8). (**M**) Quantification of hepatic triglycerides (*n* = 10). (**N**) Representative images of H&E (top) and Oil Red O staining (bottom) of liver. Scale bars, 50 μm (top) and 250 μm (bottom).

Data are shown as mean ± SEM (**A**, **C**, and **E-M**). Unpaired two-tailed Student's t-test was performed for **A**, and **F-M**. Two-way ANOVA with Sidak's multiple comparison test was performed for **C**, **J,** and **K**.

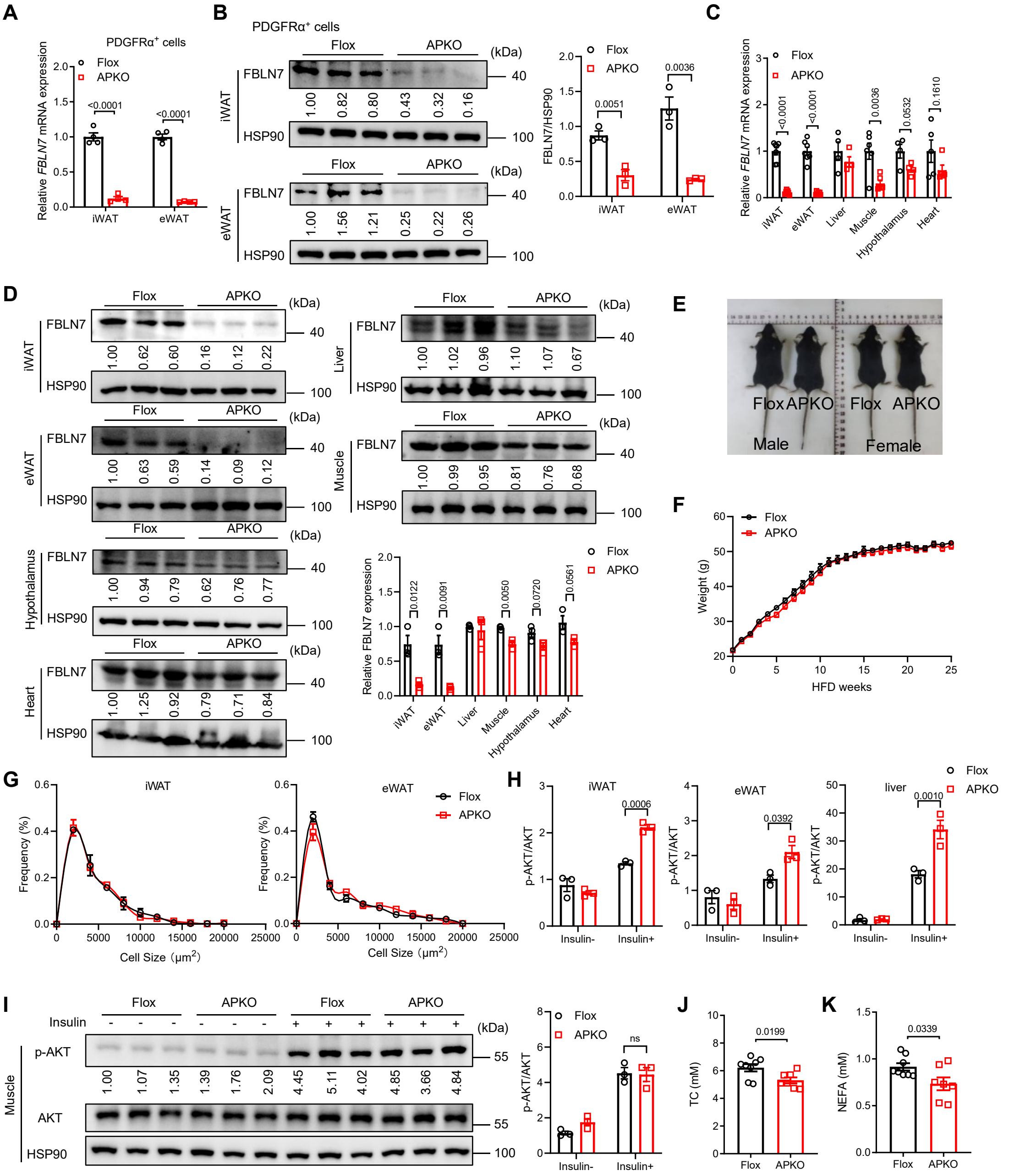

Supplemental Figure 4. ASPC-specific *FBLN7* ablation improves metabolic homeostasis

Data were compared between *FBLN7*-Flox and *FBLN7*-APKO mice. (**A**) RT-qPCR indicating *FBLN7* mRNA expression in PDGFRα+ cells from WAT ($n = 4$). (**B**) Western blot and quantification of FBLN7 in PDGFRα+ cells from WAT ($n = 3$). (**C**) RT-qPCR indicating *FBLN7* mRNA expression in different tissues ($n = 4$-7). (**D**) Western blot and quantification of FBLN7 in different tissues ($n = 3$). (**E**) Representative image of 8-week-old mice. (**F**) Body weight during 25 weeks of HFD in Flox ($n = 8$) and APKO

(*n* = 7) mice. (**G**) Quantification of adipocyte size (*n* = 3) in WAT. (**H**) Quantification of AKT phosphorylation in Figure 3H (*n* = 3). (**I**) Western blot and quantification of AKT phosphorylation in muscle after insulin or saline administration (*n* = 3). Serum TC (**J**), and NEFA (**K**) levels (*n* = 8, Flox mice; *n* = 7, APKO mice).

Data are shown as mean ± SEM (**A**-**D**, and **F-K**). Unpaired two-tailed Student's t-test was performed in **A**-**D, J,** and **K**. Two-way ANOVA with Sidak's multiple comparison test was performed for **F**. One-way ANOVA with Tukey's multiple comparison test was performed for **H** and **I**.

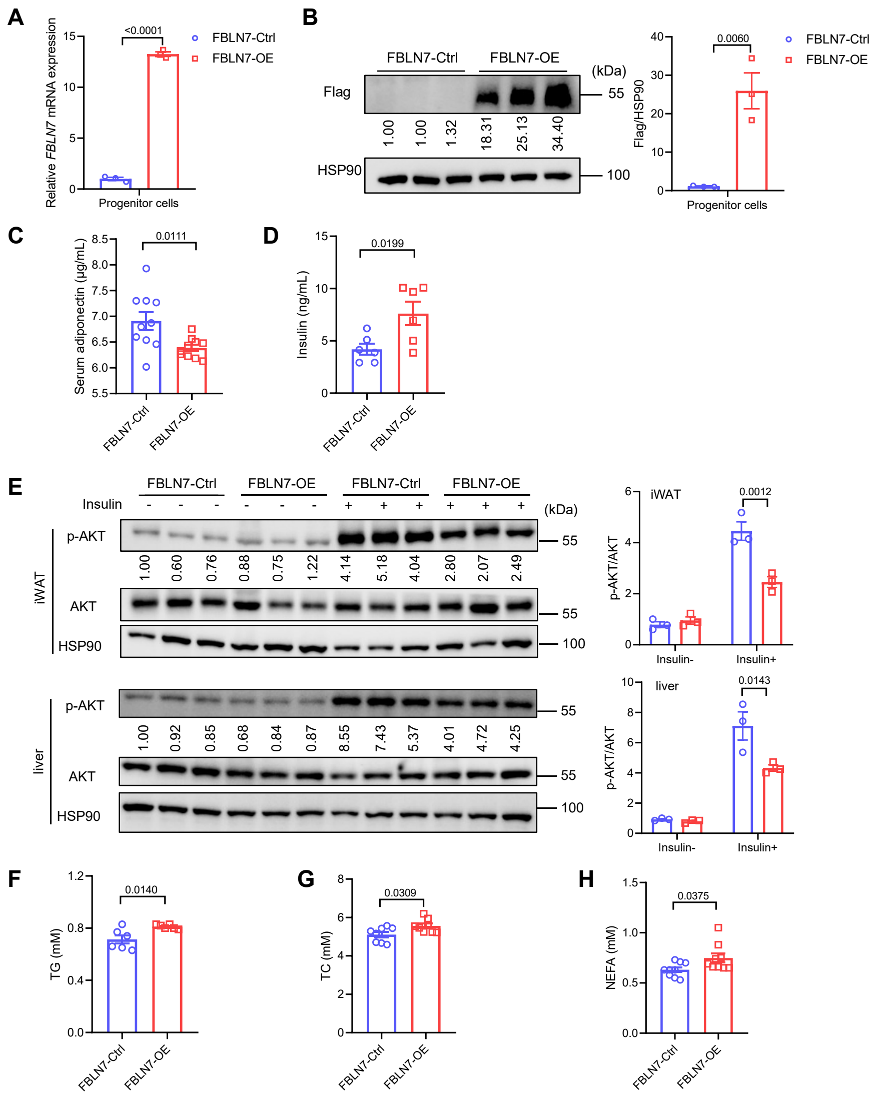

Supplemental Figure 5. FBLN7 overexpression in iWAT destroys metabolic homeostasis

Data were compared between *FBLN7*-Ctrl and *FBLN7*-OE mice. (**A**) RT-qPCR of *FBLN7* mRNA expression in PDGFRα$^+$ progenitor cells from iWAT (n = 3). (**B**) Western blot and quantification of Flag in PDGFRα$^+$ progenitor cells from iWAT (n = 3). (**C**) Serum adiponectin levels (n = 10). (**D**) Serum insulin levels (n = 6). (**E**) Western blot and quantification of AKT phosphorylation in iWAT and liver after insulin or saline administration (n = 3). (**F**) Serum TG levels (n = 6). (**G**) Serum TC levels (n = 8). (**H**) Serum NEFA levels (n = 9).

Data are shown as mean ± SEM. Unpaired two-tailed Student's t-test was performed in **A-D,** and **F-H**. One-way ANOVA with Tukey's multiple comparison test was performed for **E**.

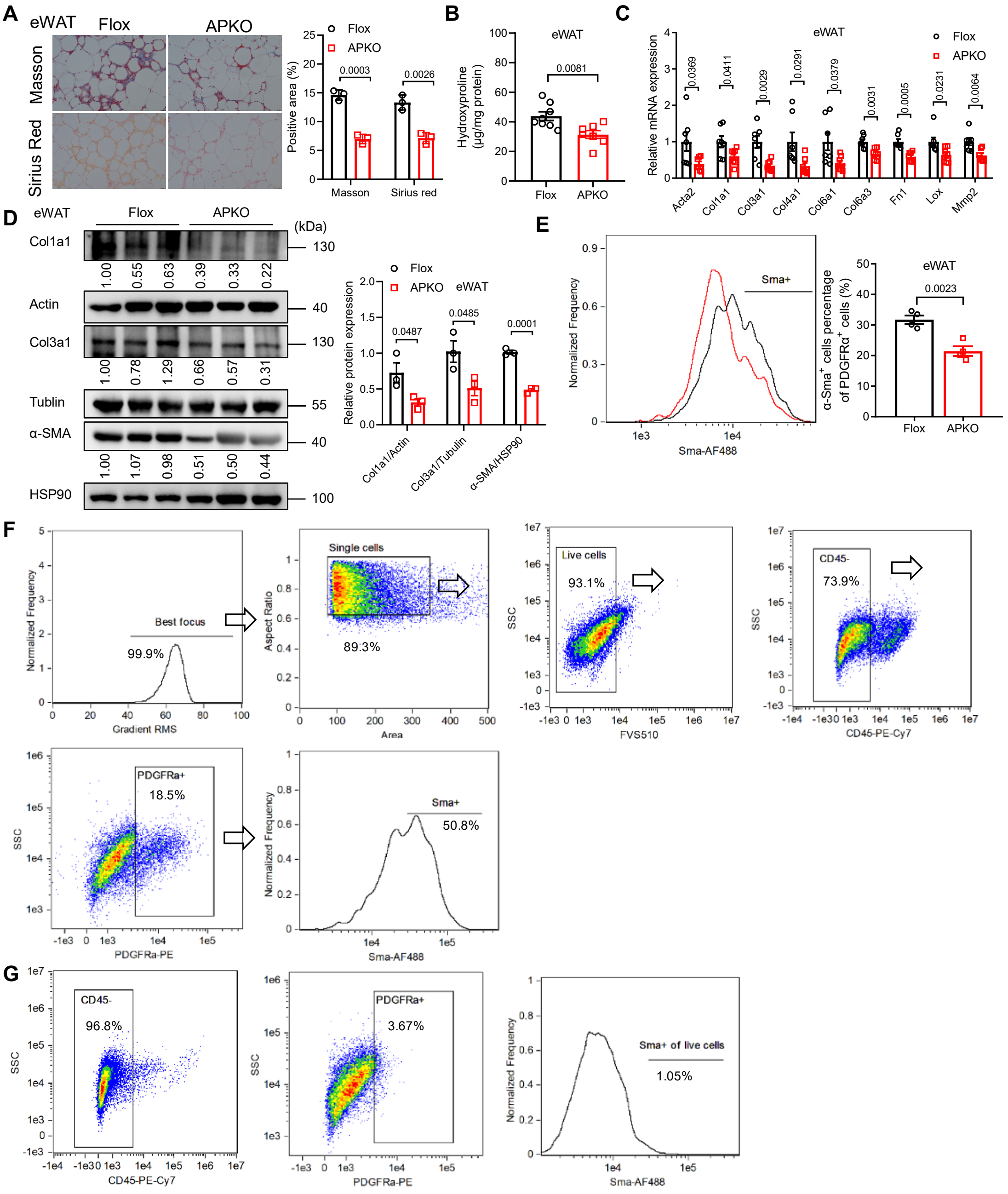

Supplemental Figure 6. ASPC-specific *FBLN7* ablation decreases obesity-related AT fibrosis

Data were compared between *FBLN7*-Flox and *FBLN7*-APKO mice. (**A**) Representative Masson's trichrome and Sirius Red staining images and quantitative analysis of eWAT (n = 3). Scale bars, 25 μm. (**B**) Hydroxyproline content in eWAT (n = 8, Flox mice; n = 7, APKO mice). (**C**) RT-qPCR indicating the mRNA abundance of pro-fibrosis genes in eWAT (n = 7). (**D**) Western blot and quantification of pro-fibrosis protein expression in eWAT (n = 3). (**E**) Representative flow cytometry analysis and

quantification of Sma^+ cells within PDGFRα^+ cells from eWAT (*n* = 4). Gating strategies (**F**) and negative control (**G**) of flow cytometry analysis.

Data are shown as mean ± SEM (**A-E)**. Two-tailed Student's t-test was performed.

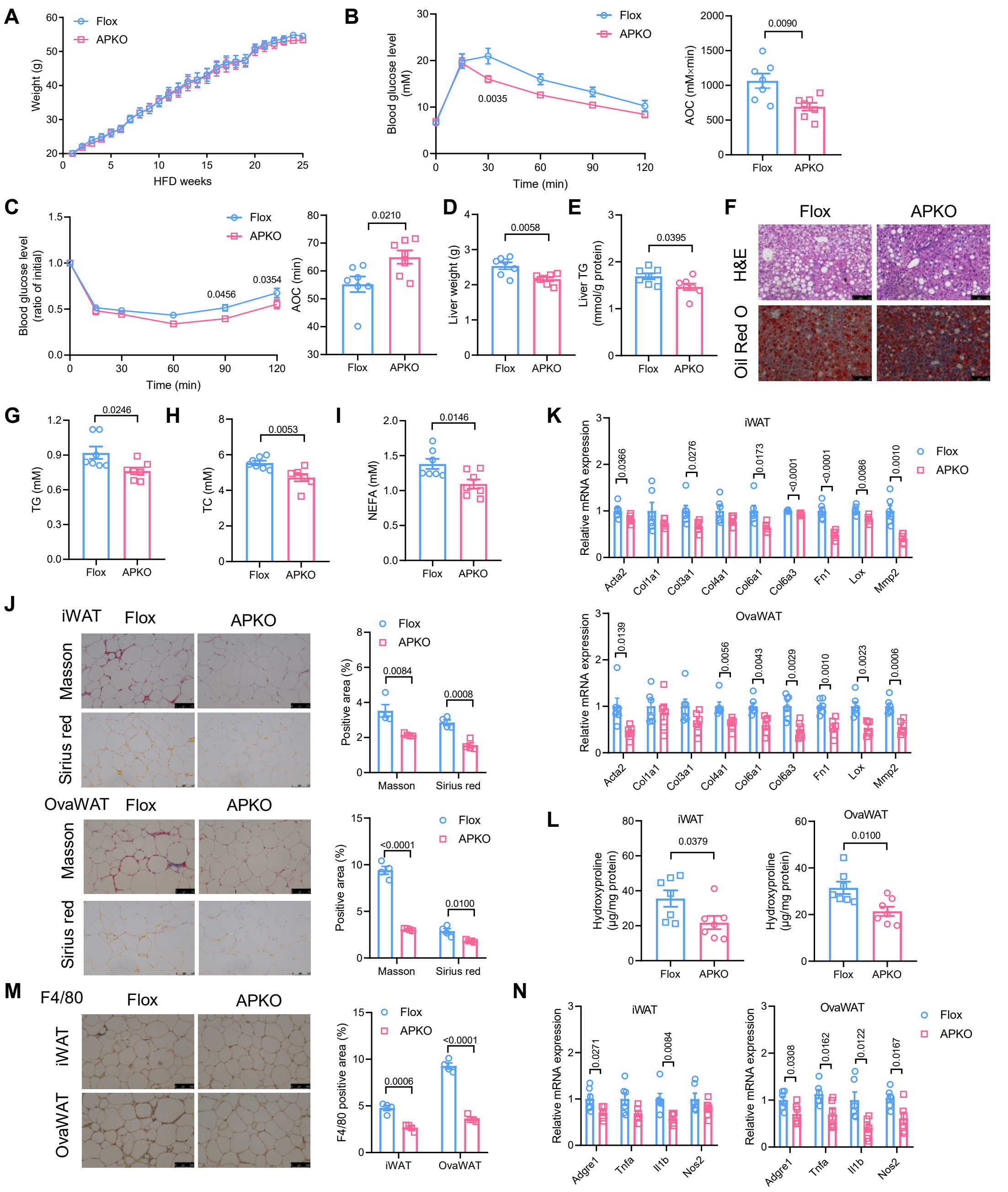

Supplemental Figure 7. ASPC-specific *FBLN7* ablation in female mice improves metabolic homeostasis and alleviates AT fibrosis

Female *FBLN7*-Flox and age-matched *FBLN7*-APKO mice were fed HFD for 25 weeks starting at 8 weeks of age. (**A**) Body weights during 25 weeks of HFD (n = 7). (**B**) GTT and AOC (n = 7p). (**C**) ITT and AOC (n = 7). (**D**) Liver weight (n = 7). (**E**) Quantification of hepatic triglycerides (n = 7). (**F**) Representative images of H&E (top) and Oil Red O (bottom) staining of liver. Scale bars, 100 μm. Serum TG (**G**), TC (**H**), and NEFA (**I**) levels (n = 7). (**J**) Representative Masson's trichrome and Sirius Red

staining images and quantitative analysis of iWAT and peri-ovarian WAT (OvaWAT) (*n* = 4). Scale bars, 100 µm. (**K**) RT-qPCR indicating the mRNA abundance of pro-fibrosis genes in iWAT and OvaWAT (*n* = 6). (**L**) Hydroxyproline content in iWAT and OvaWAT (*n* = 7). (**M**) Representative F4/80 IHC staining and quantitative analysis of iWAT and OvaWAT (*n* = 4). Scale bars, 100µm. (**N**) RT-qPCR indicating the mRNA abundance of pro-inflammatory genes in iWAT and OvaWAT (*n* = 6).

Data are shown as mean ± SEM. Two-way ANOVA with Sidak's multiple-comparison test was performed for **A-C**. Two-tailed Student's t-test was performed for **B-E**, and **G-N**.

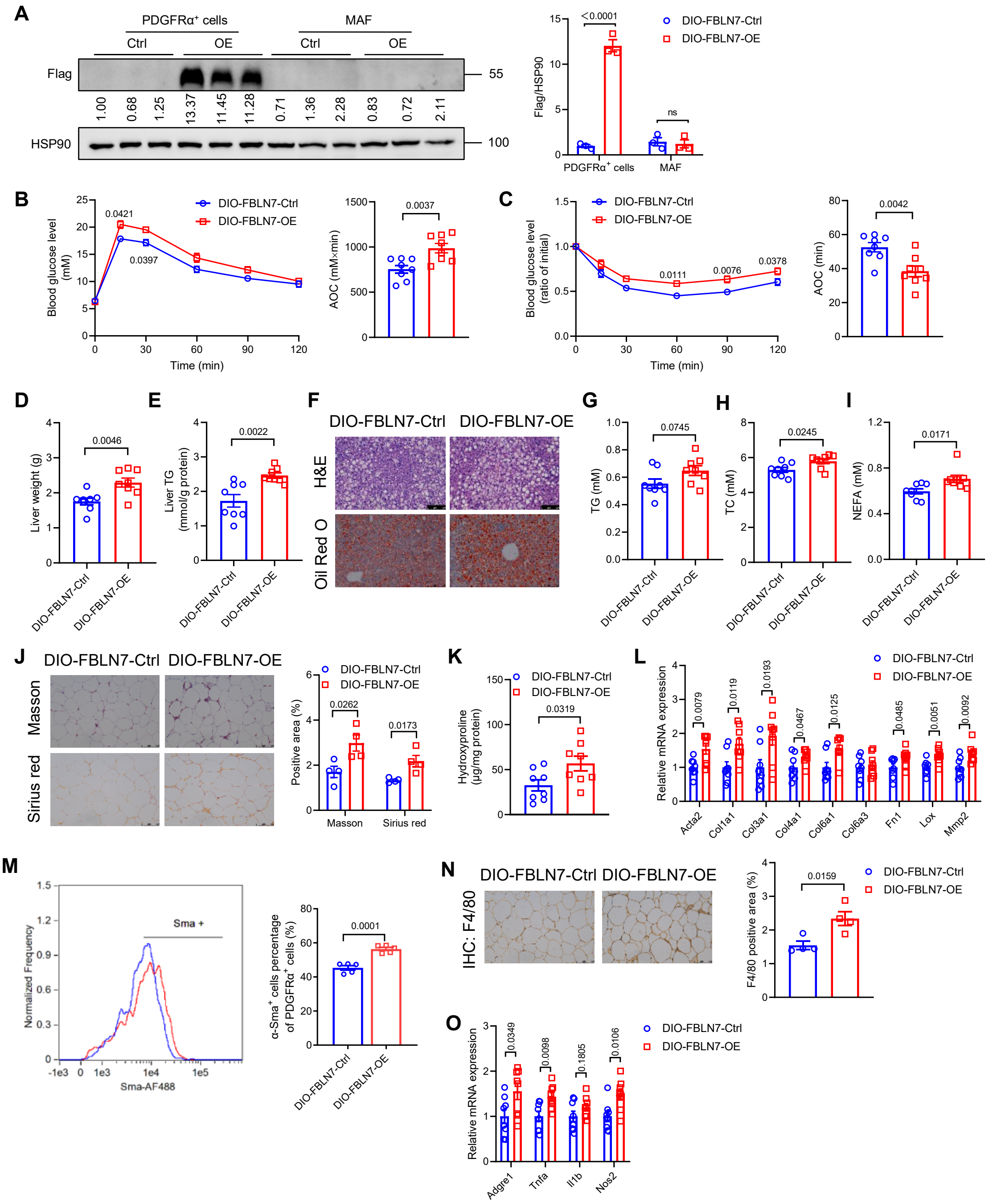

Supplemental Figure 8. ASPC-specific *FBLN7* overexpression destroys metabolic homeostasis and exacerbates AT fibrosis

At 8 weeks of age, *PDGFRα*-Cre mice received bilateral intra-iWAT injections of AAV-DIO-*FBLN7* virus to generate ASPC-specific *FBLN7* overexpression mice (DIO-*FBLN7*-OE). All mice were fed with HFD for 25 weeks. (**A**) Western blot and quantitative analysis of Flag in progenitor cells and MAF of iWAT (n = 3). (**B**) GTT and AOC (n = 8). (**C**) ITT and AOC (n = 8). (**D**) Liver weight (n = 8). (**E**) Quantification of hepatic triglycerides (n = 8). (**F**) Representative images of H&E (top) and Oil Red O

(bottom) staining of liver. Scale bars, 100 μm. Serum TG (**G**), TC (**H**), and NEFA (**I**) levels (*n* = 8). (**J**) Representative Masson's trichrome and Sirius Red staining images and quantitative analysis of iWAT (*n* = 4). Scale bars, 100 μm. (**K**) Hydroxyproline content in iWAT (*n* = 8). (**L**) RT-qPCR indicating the mRNA abundance of pro-fibrosis genes in iWAT (*n* = 8). (**M**) Representative flow cytometry analysis and quantification of Sma^+ cells within $PDGFR\alpha^+$ cells from iWAT (*n* = 5). (**N**) Representative F4/80 IHC staining and quantitative analysis of iWAT (*n* = 4). Scale bars, 100 μm. (**O**) RT-qPCR indicating the mRNA abundance of pro-inflammatory genes in iWAT (*n* = 8).

Data are shown as mean ± SEM. Two-tailed Student's t-test was performed for **A-E,** and **G-O**. Two-way ANOVA with Sidak's multiple-comparison test was performed for **B** and **C**.

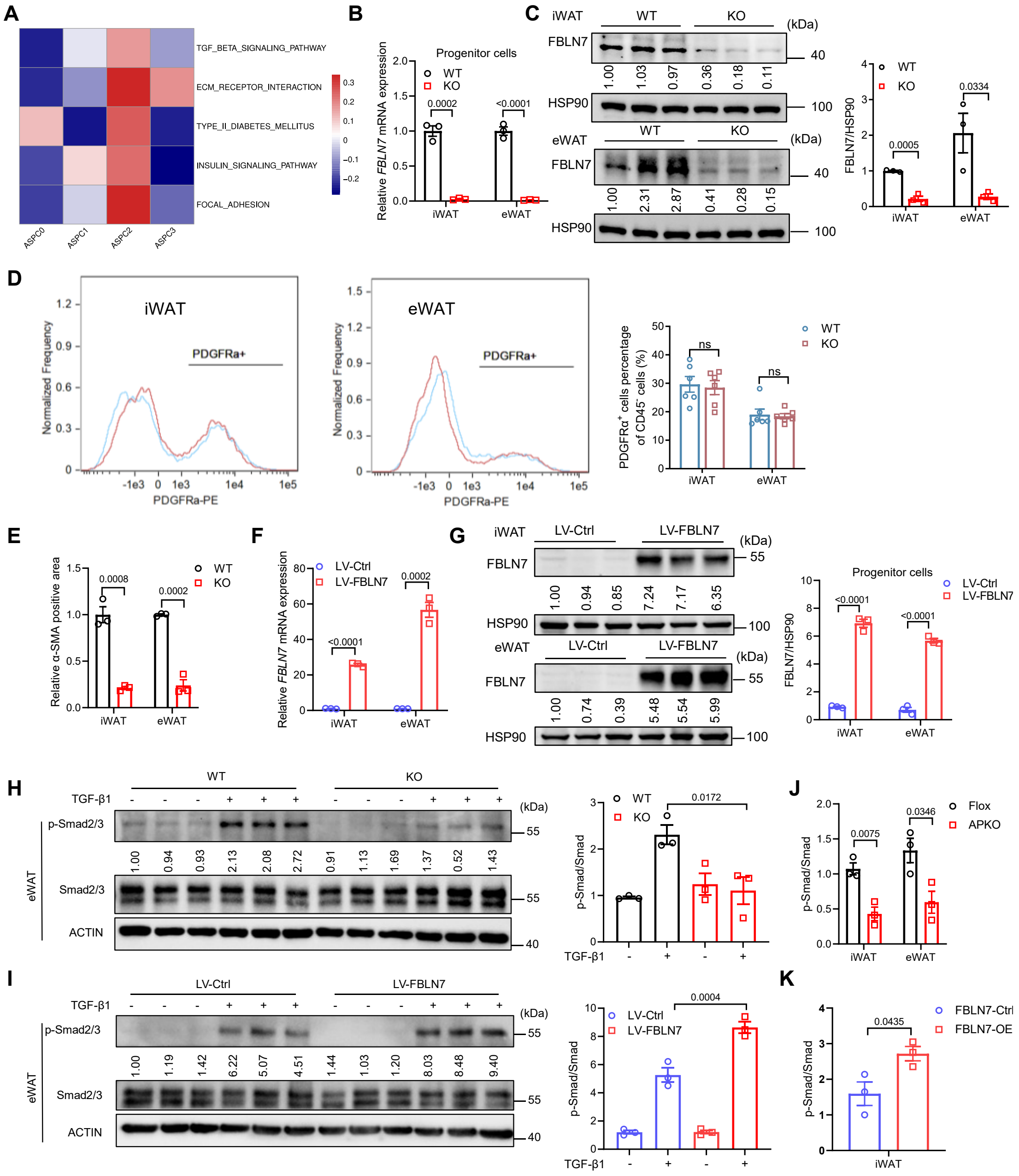

Supplemental Figure 9. FBLN7 modulates ASPCs fibrosis responses in vitro

(**A**) Heat-map of KEGG enrichment analysis for ASPCs clusters by GSVA. (**B**) RT-qPCR indicating *FBLN7* mRNA expression in progenitor cells of WAT from WT and KO mice (n = 3). (**C**) Western blot and quantification of FBLN7 in progenitor cells of WAT from WT and KO mice (n = 3). **(D)** Representative flow cytometry analysis and quantification of PDGFRα^{+} cells within CD45^{-} cells from WAT in WT and KO mice (n = 6). **(E)** Quantitative analysis of α-SMA staining in Figure 5B (n = 3). (**F**) RT-qPCR indicating *FBLN7* mRNA expression in progenitor cells infected with LV-*Ctrl* or LV-

FBLN7 (*n* = 3). (**G**) Western blot and quantification of FBLN7 in progenitor cells infected with LV-*Ctrl* or LV-*FBLN7* (*n* = 3). Western blot and quantification of p-Smad2/3 and Smad2/3 in eWAT cells from WT and KO mice (**H**) (*n* = 3), and in cells infected with LV-*Ctrl* and LV-*FBLN7* (**I**) (*n* = 3). Quantification of p-Smad2/3 and Smad2/3 in Figure 5G (**J**) (*n* = 3) and Figure 5H (**K**) (*n* = 3).

Data are shown as mean ± SEM. Two-tailed Student's t-test was performed for **B-G, J**, and **K**. One-way ANOVA with Tukey's multiple comparison test was performed for **H** and **I**.

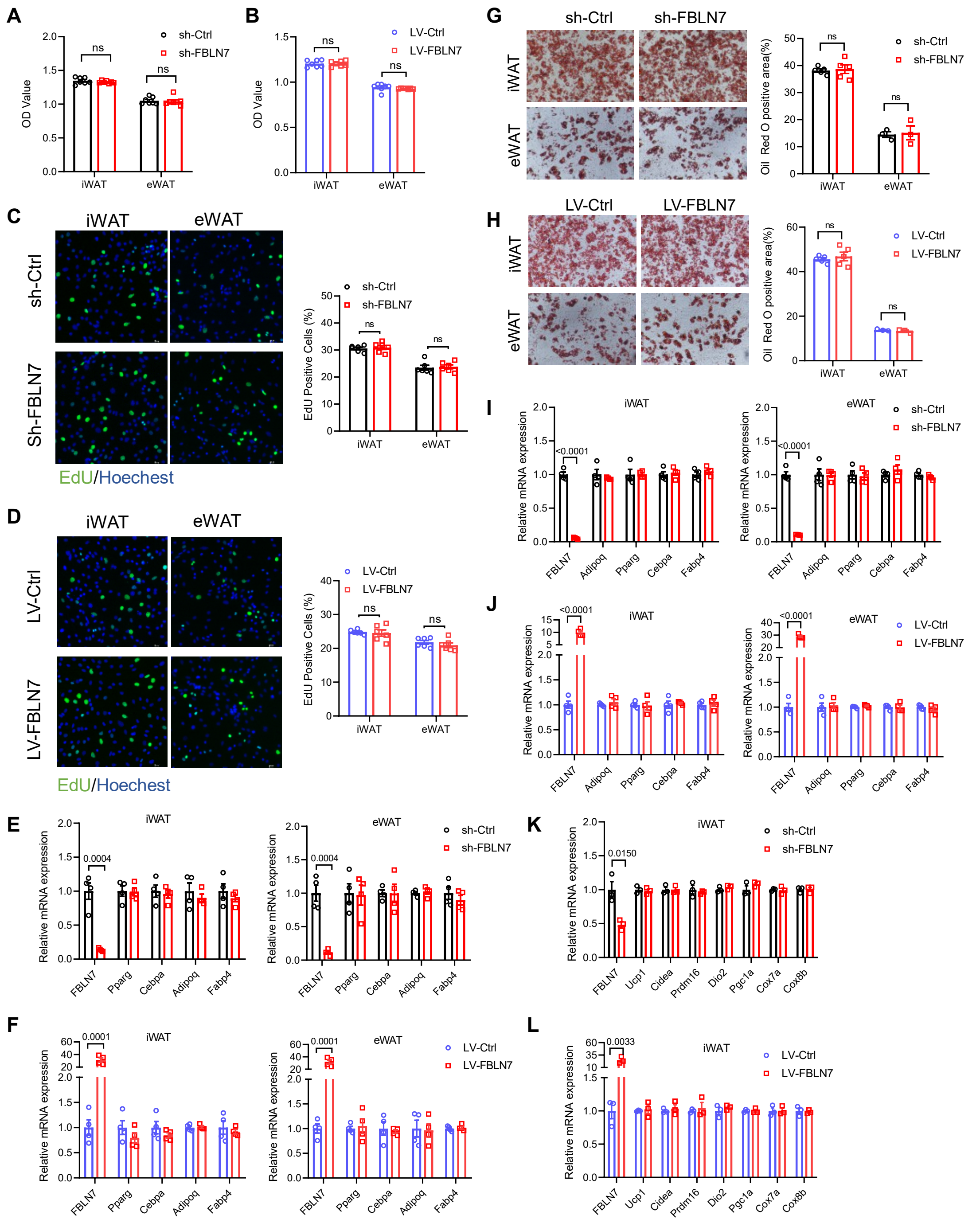

Supplemental Figure 10. In vitro experiments on differentiated mature adipocytes

CCK8 assay of progenitor cells infected with sh-*Ctrl* or sh-*FBLN7* lentivirus **(A)** (n = 7), and cells infected with LV-*Ctrl* or LV-*FBLN7* **(B)** (n = 7). Representative image of EdU staining and quantitative analysis in progenitor cells infected with sh-*Ctrl* or sh-*FBLN7* lentivirus **(C)** (n = 6), and cells infected with LV-*Ctrl* or LV-*FBLN7* **(D)** (n = 6). Progenitor cells were infected with lentivirus to knock down or overexpress *FBLN7*, and then subjected to white adipocyte differentiation tests (**F-H**). RT-qPCR indicating

the mRNA abundance of adipogenic genes in sh-*Ctrl* and sh-*FBLN7* group (**E**) (n = 4), and in LV-*Ctrl* and LV-*FBLN7* group (**F**) (n = 4). Representative Oil Red O staining images and quantitative analysis in sh-*Ctrl* and sh-*FBLN7* group (**G**) (n = 3-5), and in LV-*Ctrl* and LV-*FBLN7* group (**H**) (n = 3-5).

Progenitor cells were differentiated into white mature adipocytes, followed by lentivirus-mediated knockdown or overexpression of FBLN7 (**I** and **J**). (**I**) RT-qPCR indicating the mRNA abundance of adipogenic genes in sh-*Ctrl* and sh-*FBLN7* group (**I**) (n = 4), and in LV-*Ctrl* and LV-*FBLN7* group (**J**) (n = 4).

Progenitor cells were differentiated into beige mature adipocytes, followed by lentivirus-mediated knockdown or overexpression of FBLN7. After 48h of lentivirus infection, cells were treated with 5 μM isoproterenol (**K** and **L**). RT-qPCR indicating the mRNA abundance of thermogenic genes in sh-*Ctrl* and sh-*FBLN7* group (**K**) (n = 3), and in LV-*Ctrl* and LV-*FBLN7* group (**L**) (n = 3).

Data are shown as mean ± SEM. P values were determined by two-tailed Student's t-test.

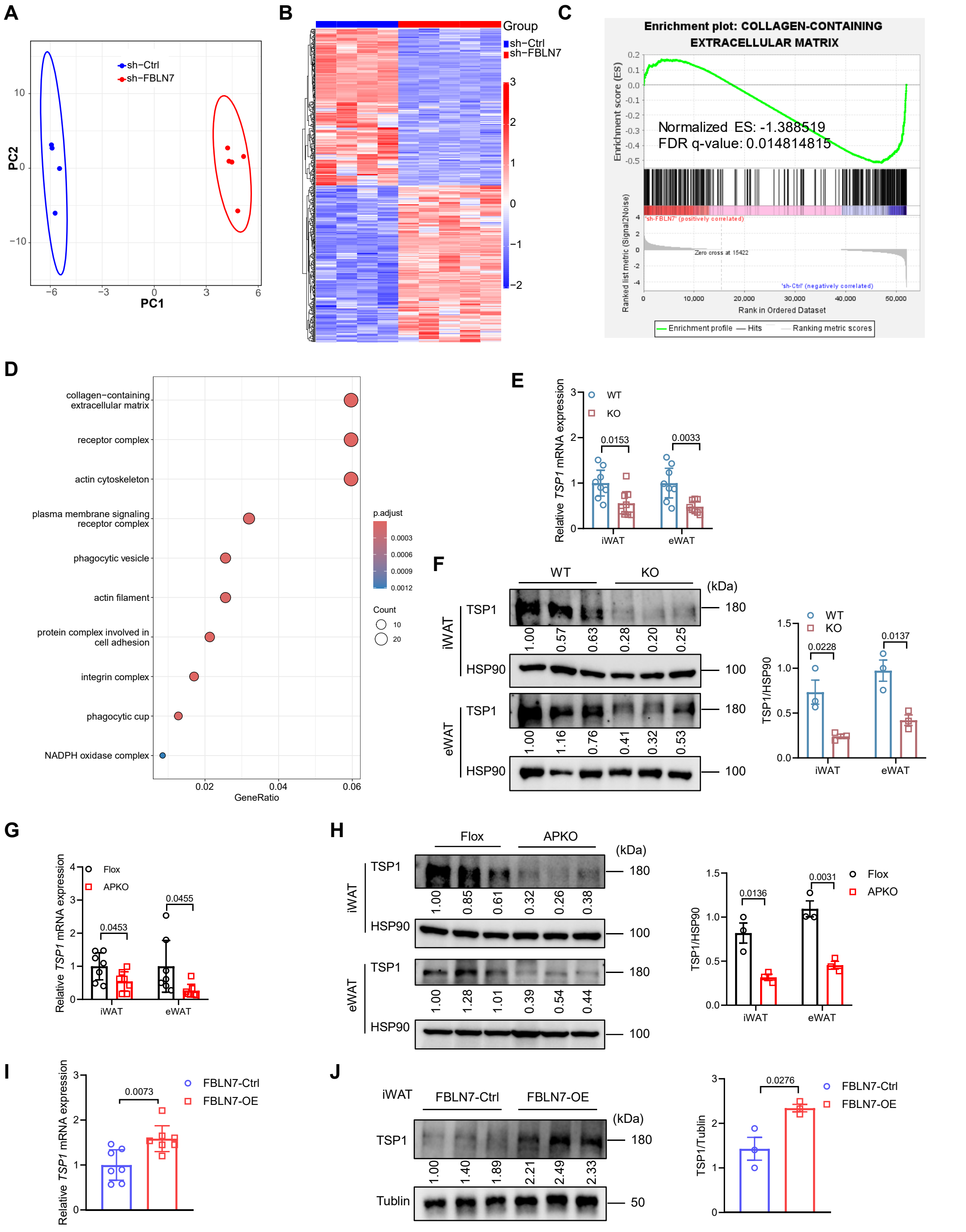

Supplemental Figure 11. RNA-seq analyses and TSP1 expression in different mouse models

(A) Principal component analysis of RNA-seq analysis in cells infected with sh-*Ctrl* (*n* = 4) or sh-*FBLN7* (*n* = 5). **(B)** Heat-map of expression profiles between the two groups. **(C)** Collagen-containing extracellular matrix pathway for enrichment plots of GSEA comparisons. **(D)** GO analysis of repressed pathways in sh-*FBLN7* compared to

the controls. (**E**) RT-qPCR indicating *TSP1* mRNA expression in WAT of WT and KO mice (*n* = 8). (**F**) Western blot and quantification of TSP1 in WAT of WT and KO mice (*n* = 3). **(G)** RT-qPCR indicating *TSP1* mRNA expression in WAT of Flox and APKO mice (*n* = 7). (**H**) Western blot and quantification of TSP1 in WAT of Flox and APKO mice (*n* = 3). **(I)** RT-qPCR indicating *TSP1* mRNA level in iWAT of *FBLN7*-Ctrl and *FBLN7*-OE mice (*n* = 7). (**J**) Western blot and quantification of TSP1 in iWAT of *FBLN7*-Ctrl and *FBLN7*-OE mice (*n* = 3).

Data are presented as means ± SEM. Two-tailed Student's t-tests were performed for **E**-**J**.

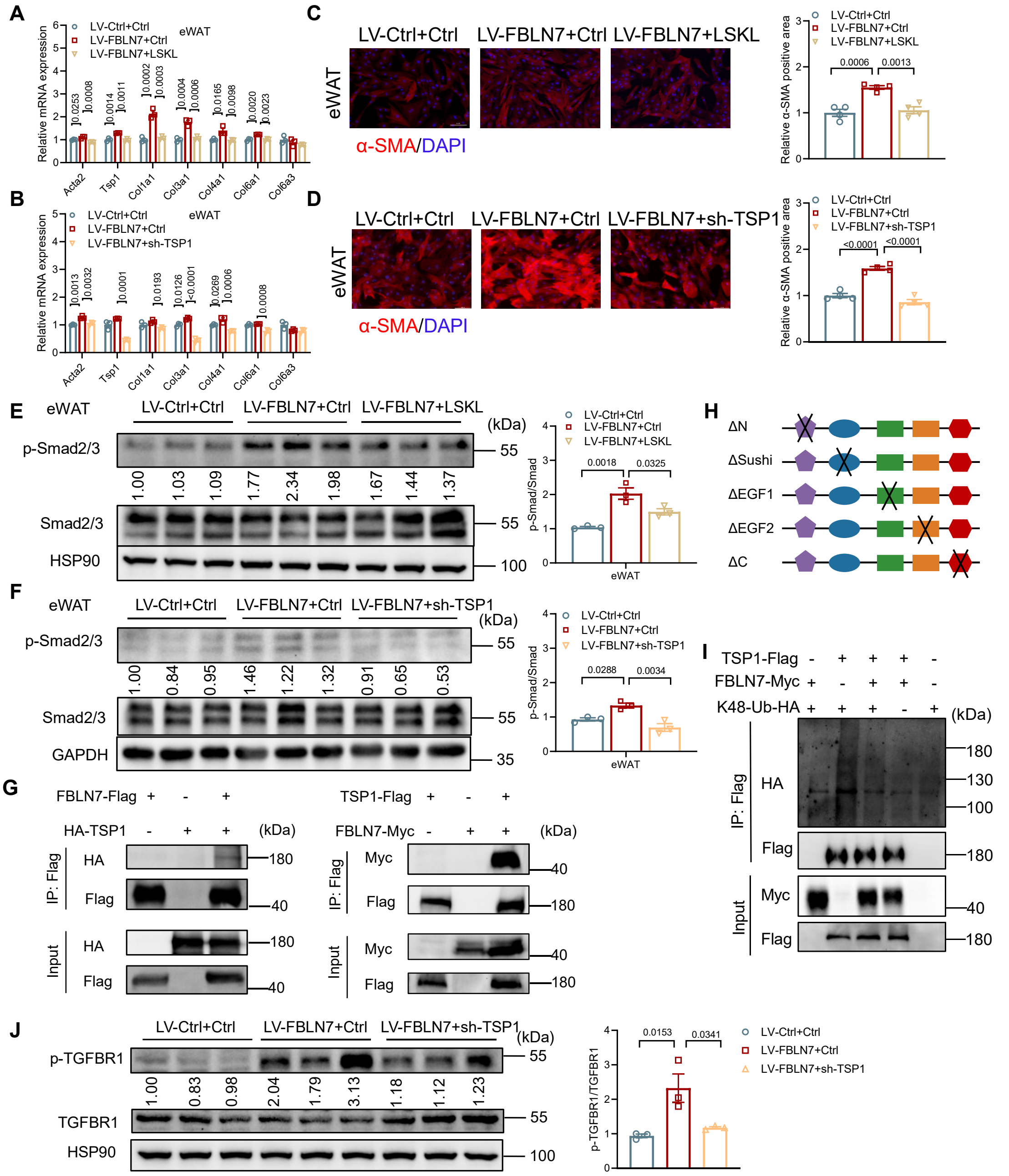

Supplemental Figure 12. FBLN7 mediates pro-fibrosis signaling via TSP1

RT-qPCR indicating mRNA abundance of pro-fibrosis genes in eWAT cells infected with LV-*Ctrl* or LV-*FBLN7* followed by LSKL treatment (**A**) (*n* = 3). or TSP1 knockdown via sh-*TSP1* (**B**) (*n* = 3). Representative immunofluorescence images and quantitative analysis of α-SMA staining (red) in in LV-*Ctrl* or LV-*FBLN7* eWAT cells after LSKL treatment (**C**) (*n* = 4), or TSP1 knockdown (**D**) (*n* = 4). Scale bars, 100 μm. Nuclei were stained with DAPI (blue). Scale bars, 100 μm. Western blot and quantification of p-Smad2/3 and Smad2/3 in LV-*Ctrl* or LV-*FBLN7* eWAT cells after

LSKL treatment (**E**) (*n* = 3), or TSP1 knockdown (**F**) (*n* = 3). (**G**) Co-IP assay in HEK293T cells between FBLN7 and TSP1. (**H**) Schematic diagram of murine FBLN7 protein and its five deletion mutants. (**I**) HEK293T cells were transfected with TSP1-Flag, FBLN7-Myc, and K48-ubiquitin-HA. Flag immunoprecipitates were analyzed for ubiquitination. (**J**) Western blot and quantification of p-TGFBR1 and TGFBR1 in LV-*Ctrl* or LV-*FBLN7* cells after TSP1 knockdown (*n* = 3).

Data are presented as mean ± SEM. One-way ANOVA with Tukey's multiple-comparison test was performed for **A-F**, and **J**.

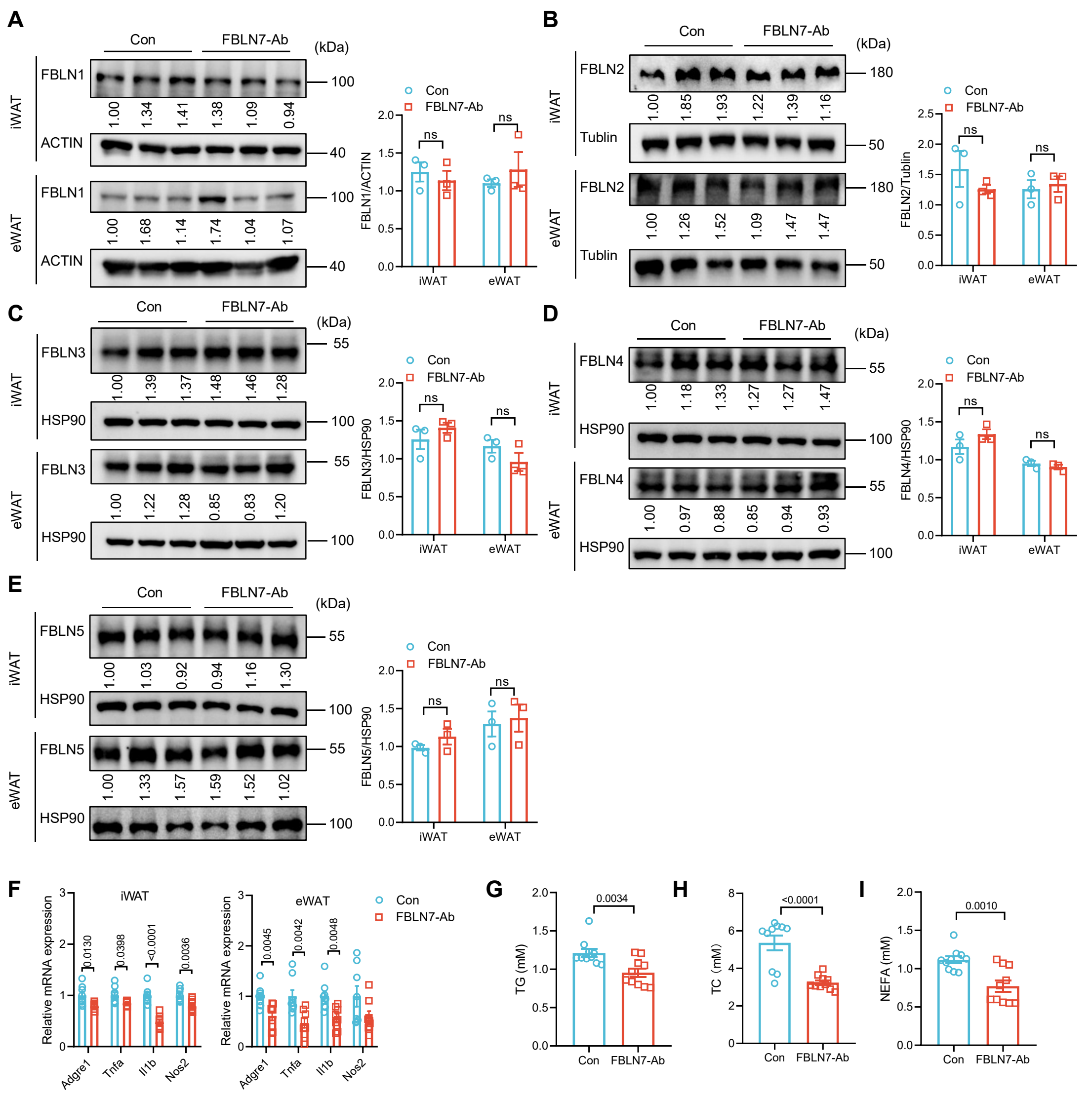

Supplemental Figure 13. FBLN7 neutralizing antibody alleviates obesity-related AT fibrosis and improves metabolic homeostasis

Data were compared between FBLN7-Con and FBLN7-Ab mice. Western blot and quantification of fibulin proteins in WAT, including FBLN1 (**A**), FBLN2 (**B**), FBLN3 (**C**), FBLN4 (**D**), and FBLN5 (**E**) (*n* = 3). (**F**) RT-qPCR indicating the mRNA abundance of pro-inflammatory genes (*n* = 8). Serum TG (**G**), TC (**H**), and NEFA (**I**) levels (*n* = 10).

Data are presented as mean ± SEM. *P* values were determined by unpaired two-tailed Student's t-test.

Supplemental Table 1. Clinical characteristics of participants (cohort 1)

**Variables**	**Normal Weight (*n* = 47)**	**Overweight/Obese (*n* = 63)**	***P* value**
Sex (men, %)	23.40	22.22	0.8837
Age (years)	46.00 ± 12.38	43.60 ± 12.58	0.3263
BMI (kg/m^2)	21.73 ± 1.72	30.39 ± 4.99	**<0.0001**
Fasting plasma glucose (mmol/L)	4.81 (4.46, 4.98)	5.11 (4.77, 5.88)	**0.0274**
SBP (mmHg)	121.24 ± 11.74	126.66 ± 16.80	0.0669
DBP (mmHg)	78.82 ± 9.00	83.33 ± 13.23	0.0518
Triglyceride (mmol/L)	0.86 (0.69, 1.08)	1.70 (1.19, 2.40)	**<0.0001**
Total cholesterol (mmol/L)	4.55 (4.07, 5.15)	4.78 (4.21, 5.39)	0.4673
ALT (U/L)	16.00 (14.00, 25.25)	21.00 (16.75, 41.25)	0.0951
AST (U/L)	21.50 (18.00, 26.75)	21.00 (17.75, 30.25)	0.5639
HDL (mmol/L)	1.14 (0.91, 1.39)	1.06 (0.84, 1.17)	0.1260
LDL (mmol/L)	2.57 (2.16, 3.06)	2.83 (2.36, 3.32)	0.6276

Normal weight: BMI < 24; Overweight/Obese: BMI ≥ 24

Data are presented as the mean ± SD, median (interquartile range) or percentage

Comparisons are done using two-tailed Student's t-test.

Prior to statistical analysis, non-normally distributed data underwent a log-transformation.

Supplemental Table 2. Characteristics of subjects with different rs147767836 genotypes (cohort 2)

**Variables**	**TT (*n* =10708)**	**TA (*n* =306)**	***P* value**
Sex (men, %)	41.55	37.91	0.2025
Age (years)	56.85 ± 7.10	57.07 ± 6.94	0.6007
Diabetes (%）	23.83	21.89	0.4325
BMI (kg/m^2)	25.02 ± 3.23	25.11 ± 3.22	0.6024
HbA1c (%)	5.70 (5.40, 6.00)	5.60 (5.40, 5.90)	0.0538
Fasting plasma glucose (mmol/L)	5.87 (5.46, 6.45)	5.78 (5.36, 6.34)	**0.0010**
2h plasma glucose after OGTT (mmol/	7.87 (6.42, 10.13)	7.49 (6.18, 10.06)	**0.0181**
Fasting insulin (mU/L)	7.29 (5.12, 10.51)	7.09 (4.94, 10.68)	0.1512
2h insulin after OGTT (mU/L)	43.81 (26.75, 70.19)	45.43 (25.58, 75.42)	0.8281
Glucose AUC	1059.15 (938.55, 1230.53)	1044 (927.53, 1196.70)	**0.0280**
HOMA-IR	1.96 (1.33, 2.94)	1.89 (1.20, 2.85)	**0.0272**
HOMA-β	59.90 (41.01, 86.40)	62.48 (40.31, 93.99)	0.5102
HOMA-ISI	0.51 (0.34, 0.75)	0.53 (0.35, 0.83)	**0.0282**
Gutt ISI	58.55 (42.20, 78.96)	60.93 (43.01, 84.65)	**0.0214**

Data are presented as the mean ± SD, median (interquartile range) or percentage
Comparisons are done using two-tailed Student's t-test.
Prior to statistical analysis, non-normally distributed data underwent a log-transformation.

Supplemental Table 3. The list of primers for mouse genotyping

	Forward primer (5'-3')	Reverse primer (5'-3')
Fbln7 -KO primer	AAGACATAAACATCAACCTCTGGC	CACATTGCTCTTGCATTTGTGTG
Fbln7- Loxp primer1	ACAGCCTTGCCTCTTGCCTTC	CCTAAGGACAACCGACACCAGAG
Fbln7 -Loxp primer2	GCTAGGACAAGGAACTAGAAGAGG	CATGGATGCCCACAAATGAC

Supplemental Table 4. The list of Assay ID for quantitative RT-PCR analysis (*Homo sapiens*)

Genes	Assay ID
RPLP0	Hs00420895_gH
FBLN7	Hs00402230_m1
ACTA2	Hs00426835_g1
COL1A1	Hs00164004_m1

Supplemental Table 5. The list of primers for quantitative RT-PCR analysis (*Mus musculus*)

Genes	Forward primer	Reverse primer
36B4	TGCCAGGACGCGCTTGT	GGCCCTGCACTCTCGCTTTC
Fbln7	GCTCGCACTGAAAGGGAGAT	GGGACCACATGCATCAACAC
Acta2	GTCCCAGACATCAGGGAGTAA	TCGGATACTTCAGCGTCAGGA
Col1a1	GTGCTCCTGGTATTGCTGGT	GGCTCCTCGTTTTCCTTCTT
Col3a1	GGGTTTCCCTGGTCCTAAAG	CCTGGTTTCCCATTTTCTCC
Col4a1	TTAAAGGACTCCAGGGACCAC	CCCACTGAGCCTGTCACAC
Col6a1	GATGAGGGTGAAGTGGGAGA	CAGCACGAAGAGGATGTCAA
Col6a3	CAGAACCATTGTTTCTCACT	AGGACTACACATCTTTTCAC
Fn1	CTGGGAACATGACCGATTTG	TTGAGGAACATGGCTTTAGGC
Lox	AGCTTGCTTTGTGGCCTTCA	CCACAGCATGGACGAATTCA
Mmp2	TAACCTGGATGCCGTCGT	TTCAGGTAATAAGCACCCTTGAA
Mmp12	ACATTTCGCCTCTCTGCTGATGAC	CAGAAACCTTCAGCCAGAAGAACC
Timp1	TTCCAGTAAGGCCTGTAGC	TTATGACCAGGTCCGAGTT
Thbs1	CCATTACAACCCAGCCCAGT	ACGTACTGGCAGTTGTCTCG
Adgre1	CCTTTGGCTATGGGCTTCCA	AGCAACCTCGTGTCCTTGAG
Tnfa	GACGTGGAACTGGCAGAAGAG	ACCGCCTGGAGTTCTGGAA
Il1b	TGGACCTTCCAGGATGAGGACA	GTTCATCTCGGAGCCTGTAGTG
Nos2	CCCAGTTGTGCATCGACCTA	ACCACTCGTACTTGGGATGC
Adipoq	GCACTGGCAAGTTCTACTGCAA	GTAGGTGAAGAGAACGGCCTTGT
Pparg	GCATGGTGCCTTCGCTGA	TGGCATCTCTGTGTCAACCATG
Cebpa	CAAGAACAGCAACGAGTACCG	GTCACTGGTCAACTCCAGCAC
Fabp4	CAGCGTAAATGGGGATTTGG	CCGCCATCTAGGGTTATGAT
Ucp1	ACTGCCACACCTCCAGTCATT	CTTTGCCTCACTCAGGATTGG
Cidea	ATCACAACTGGCCTGGTTACG	TACTACCCGGTGTCCATTTCT
Prdm16	GGCGAGGAAGCTAGCCAAA	GGTCTCCTCCTCGGCACTCT
Dio2	CAGTGTGGTGCACGTCTCCAATC	TGAACCAAAGTTGACCACCAG
Pgc1a	CCCTGCCATTGTTAAGACC	TGCTGCTGTTCCTGTTTTC
Cox7a	CAGCGTCATGGTCAGTCTGT	AGAAAACCGTGTGGCAGAGA
Cox8b	GAACCATGAAGCCAACGACT	GCGAAGTTCACAGTGGTTCC
